# The *Grounded Expertise Components Approach* in the Novel Area of Cryptic Crossword Solving

**DOI:** 10.3389/fpsyg.2016.00567

**Published:** 2016-05-03

**Authors:** Kathryn J. Friedlander, Philip A. Fine

**Affiliations:** Department of Psychology, University of BuckinghamBuckingham, UK

**Keywords:** cryptic crosswords, individual differences, Grounded Expertise Components Approach, expertise development, problem-solving, cognitive profile, aptitude, practice

## Abstract

This paper presents a relatively unexplored area of expertise research which focuses on the solving of British-style cryptic crossword puzzles. Unlike its American “straight-definition” counterparts, which are primarily semantically-cued retrieval tasks, the British cryptic crossword is an exercise in code-cracking detection work. Solvers learn to ignore the superficial “surface reading” of the clue, which is phrased to be deliberately misleading, and look instead for a grammatical set of coded instructions which, if executed precisely, will lead to the correct (and only) answer. Sample clues are set out to illustrate the task requirements and demands. Hypothesized aptitudes for the field might include high fluid intelligence, skill at quasi-algebraic puzzles, pattern matching, visuospatial manipulation, divergent thinking and breaking frame abilities. These skills are additional to the crystallized knowledge and word-retrieval demands which are also a feature of American crossword puzzles. The authors present results from an exploratory survey intended to identify the characteristics of the cryptic crossword solving population, and outline the impact of these results on the direction of their subsequent research. Survey results were strongly supportive of a number of hypothesized skill-sets and guided the selection of appropriate test content and research paradigms which formed the basis of an extensive research program to be reported elsewhere. The paper concludes by arguing the case for a more grounded approach to expertise studies, termed the *Grounded Expertise Components Approach*. In this, the design and scope of the empirical program flows from a detailed and objectively-based characterization of the research population at the very onset of the program.

## Introduction

Research on expertise development has attempted to reveal the mechanisms through which some individuals are able to show levels of performance, skill-sets, or knowledge which are reproducibly superior to that of others active in that particular domain (Ericsson and Towne, 2010). The relative contributions of deliberate practice and innate cognitive aptitude have been hotly debated [e.g., the recent special issue on expertise development in *Intelligence* (Detterman, 2014), and the recent review by Hambrick et al. (2016)], and may reflect an ideological clash between the contrasting approaches of experimental and differential psychology, with the former focusing on the general processes of skill acquisition, and the latter upon the identification of key differentiating factors in individual performance (Hambrick et al., 2014b). Studies of expertise development on both sides of the argument have tended to remain focused upon a relatively restricted range of practice-intensive domains—primarily chess, music, sport and Scrabble—and to have followed well-worn investigative paths. These have included diary/retrospective studies of practice (Ericsson et al., 1993); the *Expert-Performance Approach* (EPA—Ericsson and Ward, 2007), including paradigms based on the original de Groot chess experiments (de Groot, 1946/1965; Tuffiash et al., 2007; Ericsson and Towne, 2010); and tests of either general intelligence (“g”) itself, or a restricted set of compartmentalized sub-skills believed on *a priori* grounds to be relevant to the domain (Bilalić et al., 2007; Grabner et al., 2007; Tuffiash et al., 2007). There is a danger that, in all of these approaches, research may be based more upon preconceived, theoretical assumptions concerning the demands of the domain, or upon strongly held ideological convictions about the nature of expertise, than on grounded empirical evidence. The time is therefore right for the exploration of new domains and for a fresh theoretical and methodological perspective (Hambrick et al., 2014b, 2016).

To this end, the current paper outlines a relatively unexplored domain of investigation—British cryptic crosswords—and proposes a novel methodology, termed the *Grounded Expertise Components Approach* (GECA). This places a far heavier emphasis upon the detailed understanding and characterization of the research population and upon a holistic and empirically argued view of the demands of the performance domain, rather than a small number of isolated elements, than has hitherto been the case.

### US-style crosswords

A recent paper (Toma et al., 2014) contrasted two hypothesized cognitive drivers of proficiency (working memory (WM) capacity and strategy) in two “mind-game” domains: competitive Scrabble and national-level performance at US-style crosswords. In their introductory review, they characterized the skill-set necessary for US-style crossword solving as follows:

In contrast to competitive SCRABBLE proficiency, crossword proficiency relies on semantic aspects of language such as general word knowledge (Hambrick et al., 1999) and superior recognition for word meanings (Underwood et al., 1994). […] Unlike SCRABBLE, crossword puzzles do not require exceptional visuospatial strategies because the spatial layout of the game board is provided; therefore, visuospatial ability should not be as critical to crossword solving expertise as having an extensive understanding of word meanings. […] Semantic understanding is necessary for the process of creating a word while solving crossword puzzles; therefore, expert crossword players should primarily rely on superior knowledge of word definitions. (p. 728).

**Figure 1:**
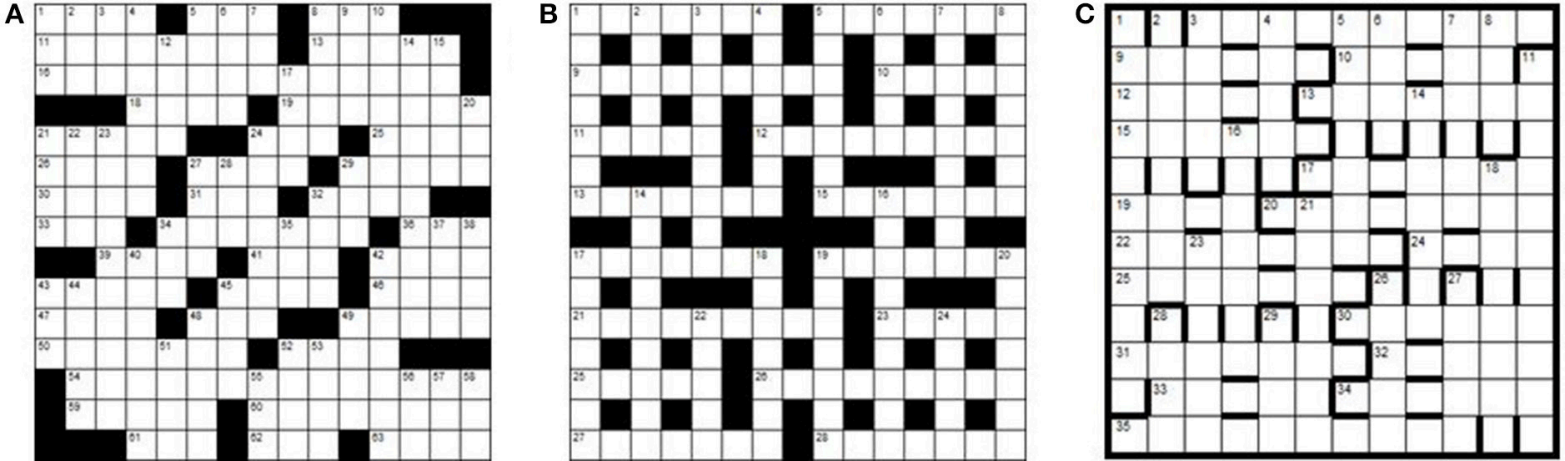
**US- and UK-style crossword grids compared**. **(A)** US-style crossword grid. **(B)** UK-style blocked crossword grid. **(C)** UK-style barred (“advanced”) grid.

Toma's characterization of crossword expertise is certainly very plausible, so far as it relates to the American crossword puzzle. At the root of the challenge set by US-style crosswords is the nature of the puzzle layout which consists of a heavily interlocking grid with fully cross-checked letters (Figure 1A). Given the constraints of the US-style grid, the creator of the puzzle (“compiler”/“setter”) often has to resort to highly obscure words, slang, brand names, sections of phrases, acronyms and even word fragments in order to populate the squares, and it is primarily this quality which determines the difficulty of the crossword (Hambrick et al., 1999; Nickerson, 2011; Sutherland, 2012). Clues are almost entirely “straight-definition” with very few exceptions; these include puns, quiz-clues and obliquely referenced clues, such as “Present time?” (8 letters), where the answer is “YULETIDE” (Shortz, 2001; Nickerson, 2011). Essentially, therefore, US crosswords may be seen as semantically cued retrieval tasks (Nickerson, 1977, 2011), requiring considerable crystallized knowledge, much of it obscure “crosswordese” [words frequently found in crossword puzzles, but very rarely in conversation (Hambrick et al., 1999; Romano, 2006)]. Additionally, good reasoning ability is hypothesized to be necessary for the evaluation of candidate responses, the deduction of entries from cross-checking letters already present in the grid (pattern recognition/word fragment completion) and the re-examination of earlier interpretations of a recalcitrant clue in order to identify misreadings of punning or ambiguous phrasing (Nickerson, 1977, 2011; Hambrick et al., 1999).

Failure to succeed in US-style crossword solving might therefore be hypothesized to arise from three causes (Hambrick et al., 1999):

1. insufficient knowledge to retrieve the target word from the preceding semantic cue;
2. inefficient retrieval strategies from long term memory;
3. weak reasoning abilities, leading to deficits in both clue interpretation and the use of ancillary information such as intersecting, cross-checking letters.

Consistent with this analysis, Toma's US-style crossword solvers were found to have significantly higher self-reported verbal SAT scores than Scrabble experts, and these scores were similar to those of undergraduates at a highly selective liberal arts college (average verbal SATs scores in the 95th percentile) acting as the control group (Toma et al., 2014). Additionally, in response to a free-text question (“What do you believe is the single most important skill you use during competition?”) participants reported their dominant cognitive abilities as “Good memory/recall” (29%) and “Mental flexibility” (26%); other key facets were “Pattern recognition” (16%), “General knowledge” (10%) and “Good vocabulary” (10%).

### British-style cryptic crosswords

British-style cryptic crosswords are very popular within the UK and in other countries with historically close ties to Britain (e.g., Canada, Ireland, Australia, New Zealand, India, and Malta). In the UK, they appear in all the daily and weekend newspapers (both broadsheet and tabloid), and also in various literary, political and cultural magazines, on the web and in specialist puzzle collections.

Although superficially similar, British-style cryptic crosswords differ from their American counterparts in fundamentally critical ways. A typical layout (Figure 1B) comprises a 15 × 15 grid with half-turn rotational symmetry in which roughly half the letters are checked by intersecting clues. The remaining spaces are filled in with “blocks”. The implications of the blocked grid design are profound: with fewer cross-checking letters, the setter no longer has to resort to the level of obscurity seen in American puzzles in order to make the entries mesh. Indeed, a former editor advises would-be setters of the (London) *Times* daily crossword:

As far as vocabulary goes, obscure words are avoided. A high percentage of the vocabulary should be familiar to a person of reasonable education and knowledge […] mostly without recourse to reference books, while commuting to work on a train, for example. (Greer, 2001, p. 52).

Challenge is therefore no longer provided by the obscurity of the semantic retrieval task. Rather, the cryptic crossword is a tricky linguistic puzzle which “plays using language as a code”, exploiting the potential ambiguity of the English language, in multiple senses, levels and contexts (Aarons, 2012, p. 224). In a cryptic clue, the apparent meaning (“surface reading”) of the clue is phrased to be deliberately misleading. The solver learns to ignore this reading, and to look instead for a non-literal interpretation of the deconstructed clue components, comprising a grammatical set of coded instructions which, if executed precisely, will lead to the correct (and only) answer. The problem lies in recognizing and cracking the code: the task of the setter, like that of a magician, is to conceal the mechanism so subtly that the solution pathway is not easily detectable at first sight.

Solvers thus have to learn how to crack a cryptic crossword: the language is an artificial creation, meaning that “no-one is a native speaker of ‘Cryptic”’ (Aarons, 2012, p. 229). Although there is general agreement that the clues have to be fairly constructed (i.e., solvable), there are no hard-and-fast guidelines as to what the rules of engagement are (Aarons, 2012), leading to an almost infinite number of innovative ways to exploit the “versatile and quirky English language” (Connor, 2013). Nevertheless, there is some consensus over a number of basic mechanism types, and a wealth of “Teach-Yourself” primers exist (Gilbert, 2001; Greer, 2001; Stephenson, 2007; Manley, 2014). The vocabulary of cryptography is often used in the titles of these works: solvers are described as “cracking the codes” and deciphering the setter's “hidden messages”. Indeed, in WWII, the British secret service MI6 recruited cryptographers to work on the Enigma Project at Bletchley Park by placing a discreet notice in the *Daily Telegraph* asking if anyone could solve the cryptic crossword in less than 12 minutes. This eventually resulted in the recruitment of six code-breakers with requisite skills from 25 applicants (Singh, 1999; Greer, 2001; Connor, 2014).

Cryptic crossword clues usually comprise two elements: a straight definition, plus the cryptic instructions for assembling the required solution—the “wordplay” (Box 1). It is not always straightforward to tell which clue element fulfills which purpose, and there is often no clear division between the two parts (Schulman, 1996; Greer, 2001; Aarons, 2012; Sutherland, 2012; Manley, 2014). Furthermore, the setter may frame the surface reading of the clue as an entirely plausible but misleading sentence, thus deliberately trapping the unwary solver in a “red herring” based on the inherent linguistic ambiguities of English (Aarons, 2012). The solver must therefore “overcome a lifetime's parsing habits” in order to avoid being sucked into the “deep structure” of the text: they must remain at the surface in order to explore other non-intuitive interpretations of the clue's components (Schulman, 1996, p. 309). In short, the cryptic is “the complicated, intellectually brooding cousin of the definitional—it had mystique and depth, it played hard to get with a capricious, whimsical air” (Brandreth, 2013).

Box 1 **Illustration of cryptic clue mechanisms.**

British-style cryptic crossword clues must be treated as a grammatical set of coded instructions. The following two clues illustrate the process:

**Clue (a)** Schulman (1996, p. 309)

**Active women iron some skirts and shirts (9)**
  The definition is “Active women” = an obliquely phrased straight definition for FEMINISTS
  The wordplay comprises: FE (iron, chemical symbol) + MINIS (plural form of a type of skirt, hence the word “some”) + TS (plural of “T,” an abbreviation for “T-Shirt”)
  The surface meaning is highly misleading; the interpretation of iron relies on a linguistic ambiguity (homonym employing different part of speech—noun, not verb).

**Clue (b)** 2013 *Times* Championship clue (http://www.piemag.com/2013/01/23/marks-mind/)

**Speciality of the Cornish side that's perfect with new wingers (5,4)**
  The definition is “Speciality of the Cornish” = CREAM TEAS
  The wordplay comprises: DREAM TEAM (“side that's perfect”) with D and M replaced by new letters on either edge (“with new wingers”).
  The surface meaning is misleadingly suggestive of football/rugby and contains some non-intuitive parsing of the components.

The algebraic/programming nature of the cryptic clue means that wordplay components may be flexibly recombined or anagrammed to form new units, e.g.:

- A+B = C (FAT+HER = FATHER)
- rev(A) = B (TRAMS → SMART)
- anag(A+B) = C (CAT+HAT = ATTACH)
- trunc(A) = B (CUTTER → UTTER)
- substring(A+B+C) = D (e.g. **Part of it 'it an iceberg (7)** = TITANIC - Moorey (2009)

Of the UK daily cryptics, the most famous is probably the *Times* crossword. Not all crosswords are equally challenging, however, and there is a widely recognized hierarchy of challenge involved (Biddlecombe, 2011; Connor, 2012; Sutherland, 2012). Difficulty in solving a standard block-style cryptic crossword is largely commensurate with the degree of concealment used by the setter in the clue mechanics, although vocabulary and clueing style can also be a factor.

One way to demonstrate expertise in cryptics is by rapid solving. Cryptic experts may be defined as those who can routinely solve a daily cryptic at the more difficult end of the spectrum in less than 30 minutes (Friedlander and Fine, 2009). As a benchmark, the average solving time required to make the elite grand final of the *Times* National Crossword Championship (TNCC) is 9–15 minutes per puzzle (Biddlecombe, 2012). Conversely, many ordinary solvers tackle easier cryptics at the same level for decades, taking an hour or more to finish (if at all).

A second way to demonstrate expertise is by successfully solving advanced cryptics (Friedlander and Fine, 2009). Advanced cryptic crosswords are found in weekend newspapers and some magazines, and are generally “barred grid” (Figure 1C). Of these, the *Listener Crossword* is the most notoriously difficult, employing a high degree of clue mechanic concealment, obscure vocabulary, grids of startling originality and a thematic challenge, often involving a number of tricky lateral thinking steps on the basis of minimal guidance. Speed is not important—solvers have 12 days to submit their solution to each *Listener* puzzle. Very few entrants achieve an all-correct year (21 in 2010; 16 in 2011; 14 in 2012^1^) and those submitting 42+ correctly (out of 52) appear on an annual roll of honor. The *Magpie*^2^, a monthly specialist magazine with 5 highly challenging advanced cryptic crosswords (and 1 mathematical puzzle) per issue, runs a similar all correct/roll of honor system, and is broadly of *Listener* standard.

Finally, for a few highly expert cryptic solvers, the ultimate challenge is to compose cryptics oneself. There are a number of clue-writing competitions [e.g., *Azed* monthly challenges in the *Observer* magazine and competitions run for *Crossword Centre* club members (Harrison, n.d.)] which attract entries from expert solvers; a few of these go on to become crossword professionals (editors or setters for local or national publications), though for most this is not their full-time occupation.

### Hypothesized cognitive demands of solving british-style crosswords

Given the above, we might therefore hypothesize that cryptic crossword solvers' skills depend less upon crystallized intelligence and cued vocabulary retrieval than those of their American counterparts, although these factors are still clearly relevant. Indeed, even if a word referenced by a cryptic clue is not known to the solver, it can often be deduced from the wordplay, and there are potentially two quite distinct avenues to the clue's solution (Coffey, 1998): the crystallized route, tapping general knowledge and vocabulary to intuit the response, perhaps using cross-checking letters; and fluid intelligence which taps the ability to “derive logical solutions to novel problems” (Hicks et al., 2015, p. 187—see also Cattell, 1963; Carroll, 1982; Kane et al., 2005) using clue components. Key cognitive abilities might therefore be hypothesized to include:

- The general capacity to analyze, reason, problem-solve and think “on one's feet,” which could reasonably be argued to draw heavily upon WM capacity and fluid intelligence (Kane et al., 2005; Shipstead et al., 2014); together with a liking for this type of cognitive challenge;
- A specific aptitude for cryptographic or mathematical thinking. The similarity of cryptic crossword clues to algebra or computer programming has been noted in passing (Manley, 2014), but has not attracted much scholarly attention. An Australian conference paper (Simon, 2004) draws a number of close analogies between solving cryptic crossword puzzles and computer programming problems, and suggests that the cryptic crossword “could one day be harnessed as one of a set of predictors of computer aptitude” claiming that “while intuition can be extremely helpful in solving crossword puzzles, it cannot take the place of clear analytical thought” (p. 302). The hunch that computer programmers and mathematicians might be particularly adept at cryptic crosswords seems to be backed up by informal membership polls undertaken by two free-membership internet-based cryptic crossword clubs (de Cuevas, 2004; Lancaster, 2005): in both surveys those engaged in STEM-based employment (Science, Technology, Engineering, Mathematics), particularly IT, were strongly represented;
- The visuospatial ability to mentally manipulate algebraic components of wordplay “fodder” (see Box 1). While many solvers use a physical jotting pad or electronic anagrammer to manipulate the letters, the ability to rapidly visualize and mentally process potentially promising combinations might be hypothesized to confer a speed advantage in solving cryptics (Minati and Sigala, 2013);
- The ability to pattern-match and, most specifically, complete word fragments provided by cross-checking letters, as already discussed for US-style crosswords (Nickerson, 1977, 2011; Hambrick et al., 1999);
- The ability to break free from familiar patterns of thought (particularly red herrings deliberately supplied by the setter) using new and unusual interpretations of clue components. The concept of “breaking the frame” of context-induced fixedness has often been associated with traditional insight problems (Davidson, 2003; Pretz et al., 2003; DeYoung et al., 2008): it is the solver's perseveration with the erroneous approach to the problem which can render it unsolvable. The authors argue elsewhere (article in preparation) that cryptic crosswords are a form of insight problem, and that flexible, divergent, “breaking frame” thinking is critical to successful solving.

The above review suggests that cryptic crosswords make wide and complex demands on their solvers, who appear to require a good all-round blend of lexical aptitude, logical/analytical thinking skills and breaking frame/lateral thinking abilities. This combination of attributes implies a certain “entry level” of ability that might in other contexts readily translate into academic success.

### Previous work on cryptic crosswords

Given the large disparity in cognitive demands and processes between the two forms of crossword (American definitional and British-style cryptic), it is essential to discriminate clearly between research undertaken in each field, since the findings from one area may not be applicable to the other, and cannot be cited uncritically as if the domains were congruent or interchangeable (Almond, 2010). Research into American-style crosswords has recently become more prolific (Nickerson, 1977, 2011; Hambrick et al., 1999; Toma et al., 2014; Moxley et al., 2015), while research into British-style cryptic crosswords has been comparatively sparse: this is mainly, one suspects, because of the separate crossword traditions of Britain and America, effectively making the subject unknown in America.

Previous research into cryptics has considered several discrete areas: exploration of the cognitive or linguistic challenges posed by cryptic clues (Forshaw, 1994; Schulman, 1996; Lewis, 2006; Aarons, 2012); the use of cryptic crosswords to preserve cognitive flexibility (“use-it-or-lose-it”) in aging populations (Winder, 1993; Forshaw, 1994; Almond, 2010); and finally a cluster of small-scale interrelated studies at the University of Nottingham, exploring individual differences in cryptic crossword solving (Underwood et al., 1988, 1994; Deihim-Aazami, 1999). Of all previous research, only the last three studies explored in any depth the question of what makes an expert solver excel in the cryptic crossword domain.

Starting from an interest in individual differences in reading ability and lexical memory, Underwood adopted the premise that cryptic crossword experts were likely to possess “particularly rich lexical networks” (Underwood et al., 1988, p. 302). In a comparison of 12 locally sourced cryptic crossword solvers with a convenience sample of 12 non-puzzlers on a battery of lexical tasks, the expectation was that the cryptic solvers, who were all of a good standard, would show particularly rapid, novel and accurate lexical data retrieval compared to the non-puzzlers. Unexpectedly, however, both groups showed very similar task performance, leading Underwood to conclude that cryptic crossword skills are “as much bound up in the cryptic puzzle codes as they are in lexical fluency” (1988, p. 306). In other words, in order to crack the cryptic challenge, solvers needed to apply problem-solving skills, in addition to possessing a good working vocabulary.

This conclusion was followed up in small-scale trials involving a convenience sample of 22 cryptic crossword enthusiasts (staff and students) from the University of Nottingham (Underwood et al., 1994; Deihim-Aazami, 1999). This population was split into “experts” (*n* = 14) and “intermediates” (*n* = 8) on the basis of their performance in solving 30 stand-alone cryptic crossword clues written by the researchers for the study. Participants were again submitted to a battery of lexical tasks, and also took the AH4 test of fluid intelligence (Heim, 1970). In a reversal of earlier findings experts out-performed the intermediates in the lexical tasks. Additionally, there was no significant difference between experts and intermediates on the AH4. From this, the researchers reasoned that success in cryptics was bound up with lexical skill and that fluid intelligence was not, after all, a factor (Underwood et al., 1994). They further hypothesized that greater exposure to cryptic crosswords over a number of years had enabled the expert solvers to broaden their vocabulary and their familiarity with cryptic clue architecture (Deihim-Aazami, 1999).

There were a number of issues with this research, however. The less-skilled group were all of student age, and had only recently commenced crossword solving, making them novices of unquantifiable future potential—“initiates” or “apprentices” according to Chi's taxonomy of proficiency (Chi, 2006), rather than experienced intermediates (“journeymen”). Additionally, neither sub-group was externally benchmarked for their performance in solving a grid-based, professionally written, publication-quality crossword of known difficulty. Both of these factors lead to doubts over the real levels of expertise present, and the assignment of participants to the correct groups. Furthermore, the experts were considerably older than the intermediates, which may have facilitated their lexical performance, as crystallized knowledge increases naturally with age (Horn and Cattell, 1967; Underwood et al., 1994). Finally, both groups scored exceptionally highly on the AH4, which was designed to be used on general populations educated only to high-school level (Deary and Smith, 2004), strongly suggesting that there were ceiling effects, given the degree-level education of the trial sample. This might explain the apparent lack of group differences in fluid intelligence.

### The expert-performance approach (EPA)

Deihim-Aazami's research (1999) contained many elements of the EPA methodological framework (Ericsson and Smith, 1991; Ericsson, 2000, 2006; Ericsson and Williams, 2007; Tuffiash et al., 2007). The aim of the EPA is to facilitate ecologically valid lab-based research in order to enable researchers to observe, analyze and capture the essence of the performance domain as the participants engage in a representative task. This task is intended to demonstrate a clearly superior performance by the expert, and elucidate potential mechanisms for this superiority (Ericsson, 2000).

The three key stages of the EPA comprise (Tuffiash et al., 2007):

- Identifying a representative task which captures the essence of expertise in the target domain. In de Groot's highly influential chess studies (de Groot, 1946/1965; Gobet et al., 2004) this consisted of two tasks: (a) identifying the best next move; (b) recalling the board layout of a briefly displayed game. The first task was intended to simulate game-play, and the second to investigate domain-specific perceptual and mnesic mechanisms. De Groot's two paradigms became classics in other “mind game” studies. For example, in the Nottingham studies, participants cold-solved 30 freestanding cryptic clues, without the aid of a grid structure or intersecting letters (Underwood et al., 1994; Deihim-Aazami, 1999). Similarly, in Scrabble, Tuffiash adapted de Groot's first paradigm, asking participants to identify the best-scoring play, when presented with a set of 12 diverse game positions that might be encountered during a highly competitive Scrabble game (Tuffiash et al., 2007).
- Observing participants engaged in this task, while collecting “process-tracing data,” often by means of a think-aloud protocol (Gilhooly and Green, 1996; Green and Gilhooly, 1996; Ericsson and Williams, 2007). Again, for Scrabble, Tuffiash recorded talk-aloud data for their participants as they debated the best next move (Tuffiash et al., 2007); and in the Nottingham trials, participants were asked to solve a further 37 isolated clues, talking aloud to explain their understanding of each clue's architecture and solution (Deihim-Aazami, 1999). Superior performance displayed by experts is argued to be directly linked to complex representations of the current task, and to be derived from a deeper and more detailed understanding of the underlying domain (Chase and Simon, 1973; Chi et al., 1981; Ericsson and Williams, 2007; Campitelli and Gobet, 2010). A systematic analysis of the material gained from process-tracing might therefore give insights into areas of comparative strength which underpin experts' superior performance (Tuffiash et al., 2007). Such data are commonly extended and enriched by identifying and experimentally manipulating additional cognitive subskills hypothesized to be key to the domain (Tuffiash et al., 2007): Tuffiash's Scrabble participants underwent an additional battery of primarily lexical tasks (Tuffiash et al., 2007); as did Deihim-Aazami's cryptic crossword participants (Deihim-Aazami, 1999), together with the AH4 (Heim, 1970);
- Accounting for the development of experts' superior performance by conducting interviews with participants, and teachers/parents where relevant, to establish key indicators of domain experience, such as starting age, key achievement milestones, and the quantity and type of practice undertaken (Tuffiash et al., 2007). This last step, and the content of the questionnaire administered, is driven by researcher conviction that the origin of high expertise in niche domains arises from extensive exposure to dedicated and structured practice regimes over at least 10 years (Ericsson et al., 1993; Ericsson, 2000). Collection of practice and experience data was therefore a feature of both the Scrabble (Tuffiash et al., 2007) and the cryptic crossword studies (Deihim-Aazami, 1999).

### Current research—the grounded expertise components approach (GECA)

The current research program could have followed the classic EPA path, by selecting representative tasks and key cognitive components from a purely theoretical standpoint, based on *a priori* detailed knowledge of the solving process. After all, the above review of hypothesized cognitive demands of British cryptics identified several promising avenues, and the Nottingham research, although small scale and conflicting in results, led the way in this domain. The current authors were reluctant, however, to impose their preconceived ideas upon the direction of the present study in this way. This reluctance was based on the conviction that one cannot conduct objective research on a niche population without first carefully characterizing this population across a large number of dimensions, leading to a grounded understanding of the drivers for participation in the field, the levels of immersion in the activity and the potential skills which are brought to it. A new approach (GECA) was therefore conceived, with the intention of providing empirical support for the direction and design of future controlled studies.

The components of the GECA comprise:

1. A wide-ranging survey characterizing the domain-specific population across a large number of dimensions, ranging from demographics, levels of participation and experience to more indirectly related areas such as participants' education, occupation and hobbies, and motivational drivers. This differs from the EPA in that the survey is not limited to the collection of practice and experience data only, and is undertaken at the very outset of the research program.
2. Analysis of the survey data to identify characteristics of both the expert and non-expert population, leading to a grounded research rationale for the design of key elements in subsequent stages of the investigation. This avoids the potential trap of confounds arising from preconceived theoretical or ideological assumptions.
3. A lab-based recording (if appropriate—see Hambrick et al., 2016) of domain-relevant performance, to elucidate both the strategic and cognitive mechanisms involved. Although this stage draws heavily on the EPA, the GECA focuses on a more naturalistic task, fully reflecting the totality of the cognitive demands of the domain, rather than a series of isolated challenges (e.g., the de Groot paradigms: on this see further the Discussion section below).
4. The identification of supplementary sub-tests, to probe specific empirically-indicated cognitive or strategic processes believed to be instrumental in distinguishing experts from non-experts within the domain: again, this flows from the initial characterization of the population.

An exploratory survey was therefore designed to capture responses of cryptic crossword solvers to a broad range of 84 questions (many with extended sub-sections), as above. Analysis of this large body of data has led to a very detailed characterization of the solving population, many aspects of which will be reported elsewhere; in the interests of brevity we report here only the key findings relevant to the design of the subsequent research program and to the establishment of the GECA as a powerful research methodology.

Although the net was cast very wide, the questionnaire was specifically set up to address the research question: “What is the nature of the cryptic crossword population in terms of their cognitive skills, motivation and expertise development?”. The hypotheses were as follows, largely flowing from the nature of cryptic solving and its cognitive demands identified earlier:

H1: cryptic crossword solvers would generally be academically able adults, given the cognitive complexity of the puzzle demands; this might imply that there is a cognitive ability threshold for entry into the domain;
H2: solvers' education and occupation would predominantly be in scientific or IT-related fields, rather than in language fields, implying that cryptics might particularly appeal to the logical and analytical thinker with an aptitude and liking for problem-solving;
H3: cryptic crossword solving regularly generates “Aha!” or insight moments, supporting the hypothesis that the cryptic clue is a classic type of insight problem through misdirection; and that this pleasurable experience is a salient driver of cryptic crossword participation;
H4: solvers would generally enjoy effortful cognitive activity in all spheres of life including work and hobbies, and that this would be an important driver of cryptic crossword participation;
H5: solving is essentially an intrinsically motivated activity, not generally undertaken for public acclaim or prize money^3^; practice/engagement levels for both expert and non-expert solvers would consequently be low and relatively unstructured compared to high profile competitive performance areas such as chess and music;
H6: cryptic solving would not normally begin in childhood, in view of the cognitive complexity of the task, but is more likely to commence in late teenage years (for US crosswords, see also Moxley et al., 2015).

## Materials and methods

### Materials

An 84-item wide-ranging questionnaire was developed and piloted. Most of the survey material was devised specifically for the study, but also incorporated the short-form “Need for Cognition” scale (Cacioppo et al., 1984) and the “Work Preference Inventory” (Amabile et al., 1994): both scales are described more fully below (Results Section). The survey was approved by the School of Science Ethics Committee, University of Buckingham.

The questionnaire was made available both on the internet through SurveyMonkey® and on paper. Respondents were recruited in two phases:

- Survey 1 involved contacting advanced cryptic crossword solvers, speed solvers and compilers at the very high-expert end of the spectrum. Respondents were sought by means of adverts circulated (i) on the *Crossword Centre* website (Harrison, n.d., a UK-based advanced cryptic crossword forum, membership approx. 950) in May 2007 and May 2008; (ii) at the TNCC in Cheltenham, England in October 2007; and (iii) with the annual *Listener* Crossword statistics, in March 2007.
- Survey 2 took place in April 2010 and invited mainstream solvers of daily block-style cryptics to take the same survey in order to obtain comparative data from non-experts. Adverts were placed on a number of websites providing a daily analysis of (and answers to) a wide range of block-style puzzles from UK newspapers; the survey was also re-advertised on the *Crossword Centre* website.

As the two questionnaires used were identical, data from the two surveys were combined and analyzed together. Participants were assigned to expertise groupings on the basis of responses to key fields within the survey (see below, “Definition of Research Groups”), regardless of survey phase.

### Participants

There were 935 responses to the surveys (S1 *n* = 257; S2 *n* = 678); however, post-consolidation reviews of the data identified a number of unworkably incomplete (*n* = 109), duplicated (*n* = 14) or spoof (*n* = 7) records, which were excluded from the analysis, leaving 805 responses in total (S1 *n* = 234; S2 *n* = 571). Participants were aged 18–84 (mean = 52.1; SD = 12.4), and males (*n* = 632, 78.5%) outnumbered females (*n* = 173, 21.5%). The majority of respondents were British (*n* = 709, 88%); the remainder were from the USA (*n* = 28, 3%), Australia (*n* = 26, 3%), Ireland (*n* = 14, 2%); Canada, New Zealand, India, Holland, France and Spain (each 1% or less).

All 805 participants responded to the initial pages of the survey; however, there was some attrition toward the end, with the lowest level of response to any section being 764. Overall reported numbers in the results section therefore vary according to the position of the question in the survey.

### Definition of research groups

A key challenge in expertise studies is that of establishing rigorous, objectively-based and externally benchmarked criteria for assigning participants to research categories (Tuffiash et al., 2007). Ranking systems such as the Elo rating in chess (Gobet and Charness, 2006) and official Scrabble tournament metrics (Tuffiash et al., 2007) are particularly valuable, and have tended to encourage research in these areas (Grabner, 2014; Hambrick et al., 2014b). No such mathematically based ranking system exists for cryptics, and alternative methods have therefore been developed (Friedlander and Fine, 2009; Fine and Friedlander, 2010) resulting in both a 2-way (Ordinary/Expert) and a 3-way (Ordinary/High/Super-Expert) categorization of participant expertise.

#### Ordinary/expert solver classification (O/E)

The Ordinary/Expert groups were designated as follows (Fine and Friedlander, 2010):

- Ordinary (O): solvers who (by self-report) normally take longer than 30 min to solve quality broadsheet cryptics. They do not usually tackle advanced cryptics, and are not successful at this form of puzzle;
- Experts (E): defined as those who can routinely solve one quality broadsheet cryptic in 30 min or less, who compile crosswords professionally, or who tackle advanced cryptics with regular success.

#### Ordinary/high/super-expert definition (O/H/S)

The above definition of “Expert” is quite broadly conceived, and does not identify world-class performance in the same way as, for example, a FIDE Elo rating of 2200 (“Candidate Master”) can do. The concept of “Super-Expert” cryptic crossword solver was therefore developed for earlier publications (Friedlander and Fine, 2009) in order to permit a more rigorous 3-way analysis of expertise, where required. In accordance with this, Super-Experts (S) were defined as those who fell into one or more of the following categories:

- Those who edit or compose cryptics professionally, on at least an occasional basis, for broadsheet or specialist publications (“Pro”);
- Those who regularly speed-solve a cryptic in < 15 min; and/or had reached the final in the annual TNCC on at least one occasion (“Speed”);
- Those who had solved 42+ *Listener* (or 48+ *Magpie*) crosswords correctly in 1 year, and were thus named on the official roll of honor (“Advanced”).

Expert (E) participants not categorized as S were designated as “High Solvers” (H), enabling data to be analyzed using a 3-way structure (O/H/S). Conceptually, these three groups are similar to Chi's “Journeyman” (O), “Expert” (H) and “Master” (S) proficiency categories (Chi, 2006).

Survey responses were strongly encouraged from those who had been tackling cryptics for at least 2 years, and preferably for 5+ years. Respondents could thus be assigned to the appropriate research group on the basis of mature performance in the field, thus avoiding possible confounds which can arise when classifying inexperienced novices of unquantifiable potential. In the event, only 11 participants with less than 2 years' experience completed the survey (1.4% of the total); of these, 9 were classified as O and 2 as H.

## Results

### Expertise ratings of the surveyed population

Solvers (*n* = 805) fell equally between O (*n* = 401, 49.8%) and E (*n* = 404, 50.2%). This high proportion of experts was attributable to recruitment methodology (particularly for Survey 1) and is not believed representative of the general level of solver expertise within the cryptic crossword solving community. Within the E group, H solvers (*n* = 225, 28.0% of overall total) outnumbered S solvers (*n* = 179, 22.2%).

As in other expertise studies (Tuffiash et al., 2007; Toma et al., 2014), the qualifying bar for the S designation was set rigorously high. Inspection of individuals coded to S revealed that many were acknowledged experts in the field, including: 49 professional setters or editors; 27 TNCC finalists, including 5 outright winners; and 111 roll of honor *Listener/Magpie* crossword solvers, of whom 31 had achieved an all-correct year. Some individuals qualified as S by virtue of two or more Pro/Speed/Advanced criteria (*n* = 52, 29% of S group). S group members were all known by name/reputation to the authors, and their skill level may be objectively verified by reference to publicly available competition statistics and compiler listings. The S category thus indisputably represents an elite body of top-class performers in the field.

### Age, experience, and gender

Demographic data were supplied by 805 respondents; key points for discussion are summarized in Table 1.

**Table 1:** **Key demographic data, by expertise group**.

**Key Demographic data**	**O**	**H**	**S**	**All Groups**
Number of respondents	401	225	179	805
Age at time of survey: Mdn (range)	54 (18–84)	53 (23–83)	54 (21–81)	54 (18–84)
Age started solving: Mdn (range)	20 (8–65)	17 (6–55)	15 (6–40)	18 (6–65)
No. of years spent solving: Mdn (range)	30 (1–62)	34 (1–64)	37 (5–66)	33 (1–66)
Gender: numbers of M/F	294/107	178/47	160/19	632/173
Gender: %F	26.7	20.9	10.6	21.5
Handedness: % left/ambidextrous	12.0	8.0	14.0	11.3
L-handedness %: S (excluding Pros)	–	–	10.8	–
L-handedness %: Pros only	–	–	22.4	–

#### Gender

The proportion of males increased with expertise (O: *M* = 294, 73.3%; H: *M* = 178, 79.1%; S: *M* = 160, 89.4%): see Table 1. There was a highly significant association between expertise and gender [$\chi_{\left(2\right)}^{2}$ = 19.01, *p* < 0.001, Cramer's *V* = 0.154]. *Post-hoc* tests using standardized residuals indicated that this was driven by female participation in the O (*z* = 2.2, *p* < 0.05) and S (*z* = −3.1, *p* < 0.01) groups, with female participation dropping from over 25% in O to around 10% in S; no other interactions were significant. The low proportion of female participants may be an artifact of sample selection (*Crossword Centre*^4^ members are predominantly male, and there is higher male participation in competitive events and professional crossword setting Balfour, 2004); however, subscriptions to the *Magpie*^5^ at the time of the survey also showed low levels of actual female participation, with membership running at 178M/10F, plus 6 couples, who solve and submit as a pair. This resulted in a female subscription level of 8% (16F out of 200, counting each member of the couple individually), comparable to our S data.

#### Handedness

Respondents were asked to supply their handedness: responses were coded as right/non-right (i.e., including ambidexterity), but will be referred to as right/left for convenience. Solvers were predominantly right-handed with overall levels of left-handedness running at 11.3% (see Table 1). Levels of left-handedness among O and H solvers fell between 8 and 12%, which would be considered low-normal within the general population (10 to 13.5% e.g., Gobet and Campitelli, 2007). S solvers show higher levels of left-handedness (14.0%); however the association between handedness and expertise was not overall statistically significant [$\chi_{\left(2\right)}^{2}$ = 3.893, *p* = 0.143, Cramer's *V* = 0.07]. Nevertheless, an analysis of the handedness of professional setters/editors (*n* = 49, all coded as S) shows a striking level (22.4%) of left-handedness; conversely other S group members (*n* = 130) now show a more conventional profile (10.8%). Reanalyzing the handedness statistics, with professionals identified separately, was statistically significant [$\chi_{\left(3\right)}^{2}$ = 8.734, *p* = 0.033, Cramer's *V* = 0.104]. *Post-hoc* inspection of the standardized residuals indicated that this was driven solely by the level of left-handedness within the Pro group (*z* = 2.3, *p* < 0.05).

#### Age and experience

Expertise groups showed very similar age profiles (O: 18–84 years of age, *Mdn* = 54; H: 23–83 years of age, *Mdn* = 53; S: 21–81 years of age, *Mdn* = 54). A Kruskal-Wallis H analysis showed no statistical difference in age between the groups [*H*_(2)_ = 0.045, *p* = 0.978].

Most cryptic crossword solvers began in their teens: 67.3% of respondents (*n* = 542) had started by the age of 20, and this rose to 88.2% by the age of 30 (*n* = 710). Starting age ranged from 6 to 65 (*Mdn* = 18), and the most popular age for commencing was 15 (*n* = 79; 9.8%) followed by 18 (*n* = 77; 9.6%). Only 21 respondents (2.6%; O: *n* = 2; H: *n* = 7; S: *n* = 12) claimed to have started solving cryptics before the age of 10; of these the majority (*n* = 20, 95%) had a parent or family member who also solved cryptics, and they learned the rules of solving from their parents (*n* = 19, 90%). There were significant group differences in starting age [*H*_(2)_ = 122.70, *p* < 0.001]: S started earliest (Range = 6–40, *Mdn* = 15) followed by H (Range = 6–55, *Mdn* = 17) and then O (Range = 8–65, *Mdn* = 20). Pairwise comparisons, using Dunn's procedure (Dunn, 1964) with Bonferroni correction for multiple comparisons, revealed statistically significant differences in starting years between all groups, all with an adjusted significance of *p* < 0.001 (effect sizes O-H *r* = 0.21; H-S *r* = 0.27; O-S *r* = 0.45).

The number of years spent solving increased with expertise, and this was broadly in line with the 2–3 year group differences in starting age (O: Range = 1–62, *Mdn* = 30; H: Range = 1–64, *Mdn* = 34; S: Range = 5–66, *Mdn* = 37). Differences were statistically significant [*H*_(2)_ = 42.81, *p* < 0.001]. Pairwise comparisons were performed, as previously. These revealed statistically significant differences in median years spent solving between O-H (*p* = 0.002, *r* = 0.14), H-S (*p* = 0.010, *r* = 0.15) and O-S (*p* < 0.001, *r* = 0.27) groups (adjusted *p*-values are presented). However, on average, members of each of the three groups had been tackling cryptics for approximately 30–40 years and were thus all highly experienced; and, regardless of group, 729 of the 805 respondents (90.6%) had been solving for 10 years or more. In common with findings in other expertise areas such as chess (Gobet and Ereku, 2014; Hambrick et al., 2014b; Campitelli, 2015), a small number of cryptic crossword solvers had achieved levels of high expertise within 5 years of starting (*H* = 9, *S* = 1). Conversely, many respondents (*n* = 57) had been solving for 45 years or more, but had remained as O.

### Practice levels

The survey collected a wide range of data relating to practice, experience and the range and difficulty of puzzles undertaken. Full details will be discussed elsewhere; the current paper presents summarized data only for key fields.

#### Hours spent solving per week

Respondents (*n* = 802) estimated how many hours they spent each week solving cryptic crosswords. Results are summarized in Table 2A.

**Table 2:** **Hours spent on (A) solving and (B) other crossword related activities**.

	**O**	**H**	**S**	**All Groups**
**(A) HOURS SPENT SOLVING/WEEK**				
Number of responses	401	225	176	802
Mean (Hours spent solving)	7.02	7.27	7.85	7.27
Median (Hours spent solving)	6	6	6	6
**(B) HOURS SPENT/WEEK ON CROSSWORD RELATED ACTIVITY**				
Number of responses	401	224	178	803
Mean (Hours other Xword activity)	1.31	1.78	3.97	2.03
Median (Hours other Xword activity)	1	1	1.5	1
Mean (Hours other Xword activity): S “Pro”			9.57	
Mean (Hours other Xword activity): S “non-Pro”			1.91	

Hours spent solving cryptic crosswords increased with expertise (O: *M* = 7.02; H: *M* = 7.27; S: *M* = 7.85); however, the median was 6 hours across all groups. This equates to approximately 45 min–1 h per day, or 1–2 blocked grid crosswords at typical solving speeds. Analysis of the difference in distribution of hours spent solving crosswords across the three groups was not statistically significant [*H*_(2)_ = 2.27, *p* = 0.321].

#### Hours spent on other cryptic crossword activity per week

Participants were also asked to estimate the amount of time spent on other crossword activities such as cryptic crossword social gatherings, blogging, consulting on-line solution pages or message boards, composing or test-solving cryptic crosswords and entering competitions. Results (*n* = 803) are summarized in Table 2B. Crossword related activity increased with expertise (O: *M* = 1.31; H: *M* = 1.78; S: *M* = 3.97); analysis of the difference in time spent on crossword related activity across groups was statistically significant [*H*_(2)_ = 47.01, *p* < 0.001]. Pairwise comparisons were performed, as previously. This revealed statistically significant differences in time spent between O-H (*p* = 0.009, *r* = 0.12), H-S (*p* = 0.001, *r* = 0.18) and O-S (*p* < 0.001, *r* = 0.28) groups (adjusted *p*-values are presented).

Much of this difference was driven by the inclusion of the 49 crossword setters/editors within the S category, who would be expected to spend considerable amounts of crossword related time each week to fulfill their professional obligations: see Table 2B. Excluding these individuals, all groups spent on average less than 2 h per week (around 20 min per day) on other crossword related activities. Differences between the groups were still statistically significant [*H*_(2)_ = 16.76, *p* < 0.001]. Pairwise comparisons revealed statistically significant differences between O-H (*p* = 0.007, *r* = 0.12) and O-S (*p* = 0.001, *r* = 0.15) alone; however comparison of H-S was no longer significant.

### Education and occupation

#### Education

Education data were supplied by 780 respondents (O: *n* = 383; H: *n* = 220; S: *n* = 177); key points for discussion are summarized in Table 3 with further discussion in the following text.

**Table 3:** **Key education data by expertise groups (with additional data for O/E split)**.

	**O**	**H**	**S**	**Total**	**E (H+S)**
**(A) LEVEL OF EDUCATION REACHED**					
Number of responses	383	220	177	780	397
Mean score (UK Gov educ level)	5.74	6.03	5.94	5.87	5.99
Mdn Level reached (6 = BA/BSc)	6	6	6	6	6
% achieving university qual	79.1%	85.0%	81.9%	81.4%	83.6%
% achieving post-grad qual	35.2%	39.5%	39.0%	37.3%	39.3%
% with PhD	10.7%	13.6%	12.4%	11.9%	13.1%
**(B) KEY EDUCATIONAL SECTORS**					
% studying STEM subjects	49.3%	50.0%	57.6%	51.4%	53.4%
% studying 'Wordsmith' subjects	25.1%	29.1%	23.7%	25.9%	26.7%
**(C) KEY STEM FIELDS**					
% studying Mathematics	13.8%^*^	19.1%	31.6%^***^	19.4%	24.7%
% studying Engineering	6.0%^*^	2.3%	1.7%	4.0%	2.0%

*,**,*** indicates significance at the 0.05, 0.01, 0.001 level.

##### Level of education reached

Respondents were asked to indicate their highest level of educational qualification either by radio button selection of standard UK options (e.g., O Level/GCSE, BA/BSc, PhD), or by free-text description, where these options were inappropriate. Data were reviewed independently by both authors, and assigned to the 8 bands of educational level (e.g., 0 = No Quals; 3 = A Level; 6 = BA/BSc; 8 = PhD) currently recognized by the UK Government (Gov.UK, 2015). An Independent Samples Mann-Whitney U test was conducted to determine whether there were differences in the distribution of educational qualification between expertise categories (O: *n* = 383; E: *n* = 397). The mean rank of E (403.76) was higher (more qualified) than for O respondents (376.76), although this trend was not statistically significant (*U* = 81,288.0, *z* = 1.77, *p* = 0.077).

Respondents as a whole were very highly educated (see Table 3A), with 81.4% (*n* = 635) having achieved a university qualification; this was higher for E solvers (83.6%, comprising H: 85.0%; S: 81.9%) than for O (79.1%), although this difference failed to achieve statistical significance [$\chi_{\left(1\right)}^{2}$ = 2.626, *p* = 0.105, Cramer's *V* = 0.058]. Many solvers had postgraduate qualifications (37.3%, typically MSc/MA or PhD) and this was again higher for E solvers (39.3%, comprising H: 39.5%; S: 39.0%) than for O (35.2%). Again, these differences failed to achieve statistical significance [$\chi_{\left(1\right)}^{2}=$ 1.365, *p* = 0.243, Cramer's *V* = 0.042]. PhDs accounted for 11.9% (*n* = 93) of all qualifications (O: 10.7%; H: 13.6%; S: 12.4%).

In view of the median age of the respondent sample (54 when completing the survey in 2007/2010, giving an approximate matriculation period of 1970–1975), these findings are exceptionally high: overall participation in UK higher education “increased from 3.4% in 1950, to 8.4% in 1970, 19.3% in 1990 and 33% in 2000” (Bolton, 2012, p. 13).

##### Main subject studied

Survey participants were asked to indicate in a free-text field the main subject they studied in their education. This information was independently reviewed by both authors and coded to 43 subject areas; after resolution of differences these were then aggregated to 17 broad subject fields (see Appendix A in Supplementary Material) within 6 educational sectors: Arts and Design; Business; Human Studies; STEM; Wordsmiths; Other (unclassifiable/too many subjects identified). Most participants specified one subject which, given the generally high level of education achieved, was considered likely to be their degree/postgraduate specialism.

Data were provided by 780 participants (O: *n* = 383; H: *n* = 220; S: *n* = 177), and were first analyzed by the 6 educational sectors. The analysis revealed a pronounced bias toward STEM subjects amongst all participants (see Table 3B). STEM subjects accounted for 51.4% of the main subjects studied, Wordsmith specialisms for 25.9% and Human Studies for 11.2%, with all other sectors being < 5% each. The proportion of STEM subjects increased with expertise (O: 49.3%; H: 50.0%; S: 57.6%). Conversely, Wordsmith subjects were least studied by S solvers (23.7%), although H studied them more than O (O: 25.1%; H: 29.1%). Overall, however, the differences in educational sector by expertise groups failed to achieve statistical significance at this aggregated level [$\chi_{\left(10\right)}^{2}$ = 12.642, *p* = 0.244, Cramer's *V* = 0.09).

A further chi-square analysis of expertise was carried out by the 17 broad subject fields. The results were highly significant [$\chi_{\left(32\right)}^{2}$ = 63.316, *p* = 0.001; Cramer's *V* = 0.201]. *Post-hoc* tests using standardized residuals indicated that this was driven by two categories: Mathematics (see Table 3C), which increased strikingly with expertise (O: 13.8%, *n* = 53, *z* = −2.5, *p* < 0.05; H: 19.1%, *n* = 42, *z* = −0.1, ns; S: 31.6%, *n* = 56, *z* = 3.7, *p* < 0.001); and Engineering, which was primarily studied by non-experts (O: 6%, *n* = 23, *z* = 2.0 *p* < 0.05; H: 2.3%, *n* = 5, *z* = −1.3, ns; S: 1.7%, *n* = 3, *z* = −1.5, ns). No other items were significant.

##### Main subject analyzed by riasec coding

Finally, educational subject data by participant (*n* = 757; O: *n* = 368; H: *n* = 214; S: *n* = 175) were assigned to “RIASEC” coding (“Holland codes”: Gottfredson and Holland, 1996; Nauta, 2010: see further Box 2) using the standard listing supplied in the “CIP to HOC” section (Classification of Instructional Program Titles to Holland Occupational Codes) of the coding manual (Gottfredson and Holland, 1996). Forty-one distinct Holland codes were applied. Twenty-three records were not coded: in most cases participants had ceased education at 14–16 years old and were unable to supply a main subject. The mapping of subject area to code was relatively straightforward, and data assignation was double-checked by a second reviewer.

Box 2 **RIASEC (“Holland” Codes)**

RIASEC (“Holland”) codes consist of a 3-letter code (e.g. “IRE,” “AES”) comprising the three RIASEC types (in order of significance) which the subject most resembles:

- “R” (Realistic: mechanical ability; works with objects, animals, plants; independent)
- “I” (Investigative: thinkers who investigate, analyze, research or problem-solve; scholarly)
- “A” (Artistic: creative, imaginative, unstructured, word-skills)
- “S” (Social: communicators, helpers, trainers; compassionate)
- “E” (Enterprising: persuaders, leaders, influencers; outgoing, energetic)
- “C” (Conventional: organizers; liking for detailed, orderly work following instructions; careful, conforming, clerical)

See further Gottfredson and Holland (1996).

The most common full Holland code assigned to participant education was “IRE” (*n* = 304, 40.2%), and S participants were particularly associated with this code (O: 38.0%; H: 35.5%; S: 50.3%). Typical educational programs associated with “IRE” are Computer Programming, Engineering, Mathematics, Physics and Analytical Chemistry. The codes “AIE” (*n* = 95, 12.5%; O: 11.1%; H: 11.7%; S: 16.6%; Modern Languages and Classics), “ASE” (*n* = 77, 10.2%; O: 11.1%; H: 13.1%; S: 4.6%; English Language and Literature) and “IRS” (*n* = 40, 5.3%; O: 5.2%; H: 4.7%; S: 6.3%; Biochemistry, Genetics, Medical Specialties) all featured prominently; the other 37 codes each accounted for < 5% of the responses. Analysis of the distribution of full Holland codes for education by Expertise groups (top 9 codes only, with the remaining codes aggregated to form a 10th group) was statistically significant [$\chi_{\left(18\right)}^{2}$ = 31.19, *p* = 0.027, Cramer's *V* = 0.144]. Inspection of standardized residuals indicated that this was driven by two values: the significantly large proportion of S participants coded to “IRE” (*z* = 2.1, *p* < 0.05) and the low proportion of S coded to “ASE” (*z* = −2.3, *p* < 0.05).

Following assignation of educational subject data to 3-letter codes, responses were analyzed by the primary RIASEC letter (see Table 4).

**Table 4:** **RIASEC analysis (primary code) of main educational field by expertise group**.

**Expertise code**	**R (%)**	**I (%)**	**A (%)**	**S (%)**	**E (%)**	**C (%)**	**Total (%)**
O	2.4	55.2	26.9	4.1	10.6	0.8	100
H	2.3	55.1	29.4	5.1	7.5	0.5	100
S	1.1	64.0	24.0	2.3	7.4	1.1	100
Total	2.1	57.2	26.9	4.0	9.0	0.8	100

This aggregation revealed a very strong bias across all groups toward “I” subjects (57.2%) generally thought to be indicative of analytical, scholarly, scientific research. “A” was evident (26.9%), but was stronger for O and H (26.9%, 29.4%) than for S (24.0%); conventional and hands-on subjects (“C,” “R”) were particularly poorly represented across the board. A chi-square analysis was not, however, significant [$\chi_{\left(10\right)}^{2}$ = 8.561, *p* = 0.574, Cramer's *V* = 0.075], although the proportion of S participants who took “I” subjects was noticeably higher (*z* = 1.2) than O (*z* = −0.5) or H (*z* = −0.4).

#### Field of paid occupation

Respondents were asked to supply their main paid occupation during their working life; teachers and lecturers were asked to state their main specialism. 780 responses were received (O: *n* = 383; H: *n* = 220; S: *n* = 177). Independent analysis of these details by both authors allowed for the allocation of respondents to 40 occupation areas based on discipline/field of work. Following resolution of differences, these were then aggregated to 23 occupational fields and thence to 7 occupational sectors: STEM, Finance, Office/Business, Wordsmiths, Performance, Manual, Other. For details, see Appendix B in Supplementary Material.

##### Analysis by occupational sectors

Data were first examined by the 7 occupational sectors (see Table 5A). The analysis revealed a pronounced bias toward STEM occupations amongst all participants. STEM accounted for 45.4% of occupations (*n* = 354), accompanied by a further 10.4% (*n* = 81) in the Financial area, where numerical/data handling skills are also assumed to be of importance. Office/Business accounted for 19.4% (*n* = 151) and Wordsmiths for 14.4% (*n* = 112), with all other sectors being < 6% each. Differences in occupational sector by expertise groups were statistically significant at this aggregated level [$\chi_{\left(12\right)}^{2}$ = 23.73, *p* = 0.022, Cramer's *V* = 0.123], and inspection of standardized residuals indicated that the key driver was Finance, where incidence increased with expertise (O: 7.8%, *n* = 30, *z* = −1.5, ns; H: 10.0%, *n* = 22, *z* = −0.2, ns; S: 16.4%, *n* = 29, *z* = 2.5, *p* < 0.05). Involvement in general Office/Business activities decreased with expertise (O: 22.5%, *n* = 86, *z* = 1.4, ns; H: 17.7%, *n* = 39, *z* = −0.6, ns; S: 14.7%, *n* = 26, *z* = −1.4, ns); and, as before, Wordsmith activity was seen predominantly in H (17.3%, *n* = 38, *z* = 1.1, ns) with S showing least involvement (S: 10.2%, *n* = 18, *z* = −1.5, ns; O: 14.6%, *n* = 56, *z* = 0.1, ns). However, these latter drivers failed to achieve statistical significance.

**Table 5:** **Percentage involvement in occupational categories, by expertise group**.

	**O**	**H**	**S**	**Total**
Number of responses	383	220	177	780
**(A) BY 7-WAY ANALYSIS OF OCCUPATIONAL SECTOR**				
Finance	7.8%	10.0%	16.4%^*^	10.4%
STEM (including Medicine)	45.2%	42.7%	49.2%	45.4%
Office/Business	22.5%	17.7%	14.7%	19.4%
Wordsmiths	14.6%	17.3%	10.2%	14.4%
Performance	3.4%	4.5%	1.7%	3.3%
Manual	1.6%	2.7%	0.6%	1.7%
Other	5.0%	5.0%	7.3%	5.5%
	100%	100%	100%	100%
**(B) WITH DETAILED ANALYSIS OF FINANCE/STEM AREAS**				
Actuarial/Economics	1.6%	3.6%	3.4%	2.6%
Banking/Accountancy	6.3%	6.4%	13.0%^*^	7.8%
Science	5.0%	7.3%	5.6%	5.8%
Technology/IT	20.6%	24.5%	31.6%^*^	24.2%
Engineering	6.0%^**^	0.9%^*^	1.1%	3.5%
Mathematics	6.5%	3.6%	8.5%	6.2%
Medicine	7.0%	6.4%	2.3%	5.8%
Not STEM/Finance	47.0%	47.3%	34.5%^*^	44.2%
	100%	100%	100%	100%

*,**,*** indicates significance at the 0.05, 0.01, 0.001 level.

Data relating to key areas of interest (STEM and Finance) were explored in finer detail (see Table 5B). By far the largest category, accounting for 24.2% of responses (*n* = 189) across all groups, was Technology/IT. A chi-square analysis of Table 5B data was highly significant [$\chi_{\left(14\right)}^{2}$ = 46.08, *p* < 0.001; Cramer's *V* = 0.172]. *Post-hoc* tests using standardized residuals indicated that this was driven by four areas: Banking/Accountancy, which increased with expertise (O: 6.3%, *n* = 24, *z* = −1.1, ns; H: 6.4%, *n* = 14, *z* = −0.8, ns; S: 13.0%, *n* = 23, *z* = 2.5, *p* < 0.05); Engineering, predominantly pursued by O solvers (O: 6.0%, *n* = 23, *z* = 2.7, *p* < 0.01; H: 0.9%, *n* = 2, *z* = −2.0, *p* < 0.05; S: 1.1%, *n* = 2, *z* = −1.7, ns); and Technology/IT which increased with expertise (O: 20.6%, *n* = 79, *z* = −1.4, ns; H: 24.5%, *n* = 54, *z* = 0.1, ns; S: 31.6%, *n* = 56, *z* = 2.0, *p* < 0.05). Thus, nearly $\frac{1}{3}$ of S solvers pursued a career in the IT field, compared to $\frac{1}{5}$ of O solvers. The aggregated category (Not STEM/Finance) was also significant, with S showing a significantly lower proportion of job activity outside these two areas (O: 47.0%, *n* = 180, *z* = 0.8, ns; H: 47.3%, *n* = 104, *z* = 0.7, ns; S: 34.5%, *n* = 61, *z* = −2.0, *p* < 0.05).

##### Occupation analyzed by RIASEC coding

Participant occupation data (*n* = 769; O: *n* = 380; H: *n* = 217; S: *n* = 172) were assigned to Holland codes by an independent coder, using the standard listing supplied in the “DOT to HOC” section (Dictionary of Occupational Titles to Holland Occupational Codes) of the coding manual (Gottfredson and Holland, 1996). Sixty-one distinct Holland codes were assigned in this process. Eleven participant records were not coded: in these cases respondents had either not worked (e.g., through ill-health) or were unclassifiable.

Following double-checking by both authors and resolution of differences, the full Holland codes were scrutinized. The code “IRE” was again most commonly found, being assigned to 155 participants overall (20.2%), and this increased with expertise (O: 18.7%; H: 21.2%; S: 22.1%). Occupations typically assigned to this code included geologists, statisticians, mathematics teachers/lecturers, software engineers and science teachers/lecturers. The codes “IER” (*n* = 56, 7.3%; O: 7.6%; H: 7.8%; S: 5.8%; engineers, financial consultants, systems analysts), “AIE” (*n* = 51, 6.6%; O: 6.6%; H: 8.8%; S: 4.1%; English teacher, writers/editors), “IRC” (*n* = 45, 5.9%; O: 5.3%; H: 5.5%; S: 7.6%; computer programmers) and “CSI” (*n* = 30, 3.9%; O: 3.2%; H: 3.7%; S: 5.8%; accountants) were also prominent; the other 56 Holland codes each accounted for < 3.9% of the responses. Analysis of the distribution of full Holland codes by expertise (top 19 codes only, with the remaining codes aggregated to form a 20th group) failed, however, to achieve statistical significance [$\chi_{\left(38\right)}^{2}$ = 34.72, *p* = 0.622, Cramer's *V* = 0.150).

Responses were also analyzed by the primary RIASEC letter and compared with indicative norms for the 2010 US workforce (McClain and Reardon, 2015): see Table 6.

**Table 6:** **RIASEC analysis of main occupational field by expertise group in comparison with US workforce norms**.

	**R (%)**	**I (%)**	**A (%)**	**S (%)**	**E (%)**	**C (%)**	**Total (%)**
O	3.9	41.8	12.9	17.9	16.6	6.8	100
H	4.6	45.6	15.2	12.4	15.7	6.5	100
S	4.1	45.9	7.6	16.3	16.3	9.9	100
Total	4.2	43.8	12.4	16.0	16.3	7.4	100
% US							
Workforce 2010	27	10	2	24	20	17	100

Overall scores again showed a strong bias toward “I” activities, accounting for 43.8% of the responses (O: 41.8%; H: 45.6%; S: 45.9%), compared to 10% in the 2010 US population norms (McClain and Reardon, 2015). Given the broadly comparable economic and technological profiles of the US and the UK, this appears considerably higher than might be expected. Employment in “I” fields typically involves relatively small numbers of largely STEM-based occupations involving highly-qualified individuals (McClain and Reardon, 2015).

“A” occupations (12.4%; publishers, journalists, writers, English teachers) were also considerably more prominent than US population norms (2%), and this Wordsmith activity was once more higher for H participants, with S being particularly low. While a chi-square analysis of primary RIASEC codes by expertise failed to achieve overall significance [$\chi_{\left(10\right)}^{2}$ = 10.05, *p* = 0.436, Cramer's *V* = 0.081), the comparatively low proportion of S occupied in “A” careers approached significance (S: 7.6%, S = 13, *z* = −1.8).

Code “S” was more prominent for survey occupation data than for education, and tended to reflect occupational team-building, committee and communications skills: for example, “SEC” (*n* = 29, 3.8%; e.g., civil servant) and “SER” (*n* = 22, 2.9%; e.g., company board director). Careers assigned to “R” tend to involve high levels of employment in relatively low-grade practical or mechanical tasks (McClain and Reardon, 2015), and were poorly represented among our survey population. Careers coded to “C” were more common for S (*z* = 1.2) than for O (*z* = −0.4) or H (*z* = −0.5), reflecting the larger proportion of accountants in this group (“CSI”).

##### Occupation complexity

As part of the Holland coding process, occupation complexity was also recorded, using the Cx rating score in the “DOT to HOC” section of the coding manual (Gottfredson and Holland, 1996). This rating reflects the cognitive complexity of work demands (for the calculation algorithm, see Gottfredson and Holland, 1996, p. 723). Holland Cx scores range from < 40 to >80; a Cx rating of 65 or higher is associated with a college degree and 4–10 years of “On-Job-Training” (Reardon et al., 2007).

Survey respondents were engaged in cognitively complex jobs, with the mean and median scores for all groups being close to 70: see Table 7. Over half the respondents (53.8%) fell into the 70–79 band. Typical career options in the 60–69 band include secondary school teachers, middle-ranking civil servants and journalists; and in the 70–79 band lawyers, physicians, software engineers, company directors and university faculty staff. There were 151 teachers: 102 were predominantly secondary school specialists (Cx = 66); 49 were university faculty members (Cx = 74), including 9 at UK professorial level (Cx = 78).

**Table 7:** **Occupation complexity score (Cx) by expertise category**.

	**O**	**H**	**S**	**Total**
Number of responses	380	217	172	769
Mean Cx score	69.2	69.4	69.7	69.4
Mdn Cx score	70	70	70	70
**% BY COMPLEXITY BAND**				
40–49	0.5%	1.4%	–	0.7%
50–59	4.7%	6.0%	1.7%	4.4%
60–69	41.8%	35.5%	42.4%	40.2%
70–79	52.1%	56.2%	54.7%	53.8%
80+	0.8%	0.9%	1.2%	0.9%

Job complexity increased slightly with expertise (O: *M* = 69.2; H: *M* = 69.4; S: *M* = 69.7) and there were fewer S participants in the two lower complexity bands; however, job complexity did not differ significantly across expertise groups [*H*_(2)_ = 1.230, *p* = 0.541).

### “Need for cognition” and hobbies

#### “Need for cognition”

The short-form “Need for Cognition” (NFC) scale seeks responses to 18 statements relating to a person's tendency to seek out, engage in and enjoy effortful thinking (Cacioppo et al., 1984; von Stumm and Ackerman, 2013). Sample questions include (both reverse coded): “*I like tasks that require little thought once I've learned them*” and “*Thinking is not my idea of fun.*” Respondents (*n* = 764; O: *n* = 377; H: *n* = 212; S: *n* = 175) rated each statement on a 5-point Likert scale (1 = “Completely Disagree”; 5 = “Completely Agree”). Full details of the results obtained will be discussed elsewhere; the current paper presents summarized data only.

Scores were corrected for reverse coding and averaged by participant to produce an individual NFC score. Overall, respondents showed mean NFC levels significantly in excess of the mid-point, 3: *t*_(763)_ = 36.55, *p* < 0.001, *d* = 1.32. Indeed, no expertise group means fell below 3 on any of the individual 18 statements. NFC increased with expertise (O: *M* = 3.71, *Mdn* = 3.78; H: *M* = 3.77, *Mdn* = 3.78; S: *M* = 3.79, *Mdn* = 3.83) but differences were not statistically significant [*H*_(2)_ = 3.319, *p* = 0.190]. A Mann-Whitney *U* test was also conducted to determine whether there were differences in the distribution of NFC scores between broad expertise categories (O: *n* = 377; E: *n* = 387). The mean rank of E (396.54) was higher (greater NFC) than for O respondents (368.09) and this approached statistical significance (*U* = 78,383.5, *z* = 1.783, *p* = 0.075).

In a separate question (reported later in **Table 12**), participants were asked to rate suggested motivators for their engagement with cryptic crossword solving. Full details of these motivators will be discussed elsewhere; however the wording of one suggested motivator is strongly reminiscent of the NFC: “*My brain constantly demands to be engaged in intellectual pursuits in all I do.”* Responses (*n* = 786; O: *n* = 388; H; *n* = 221; S: *n* = 177) rated this statement on a 5-point Likert scale, as above. Scores increased with expertise (O: *M* = 2.72, *Mdn* = 3; H: *M* = 3.2, *Mdn* = 3; S: *M* = 3.36, *Mdn* = 4) and differences were statistically significant [*H*_(2)_ = 37.79, *p* < 0.001]. Pairwise comparisons were performed as above. These revealed statistically significant differences between O-H (*p* < 0.001, *r* = 0.18) and O-S (*p* < 0.001, *r* = 0.23) groups (adjusted *p*-values are presented). The difference between H-S was not statistically significant.

### Hobbies

Participants were also asked about their hobbies outside cryptic crosswords. Data were collected in two parts: the first collected details of levels of engagement with other mind game activity (e.g., chess, Sudoku, Scrabble, non-cryptic crosswords); the second asked for free-text details of any other significant hobby activity. Data from these two sources were segmented, coded and combined to provide a rounded picture of cryptic crossword enthusiasts' leisure-time activities; mind game data were included only for those participants who engaged “regularly” with these activities. Final consolidated data related to 718 participants (O: *n* = 353; H: *n* = 197; S: *n* = 168) who participated in a total of 2687 hobbies (responses per participant, Range = 1–14; *M* = 3.74, *Mdn* = 3).

Sixty-two participants (8%) indicated 8% (*n* = 62) indicated that they had no important hobbies other than cryptic crosswords, or left this field blank (O: *n* = 31, 8%; H: *n* = 22, 10%; S: *n* = 9, 5%).

#### Hobbies by fields of interest

Hobbies were coded to hobby areas by an independent coder; these were then aggregated to 10 broad hobby sectors (see Table 8A), and three researchers (including the authors) then reviewed and agreed all codings. Overall, hobby replies (*n* = 2687) showed a pronounced bias toward cognitively challenging pastimes: 39.9% (*n* = 1073) related to other mind games (9.1% Sudoku, 4.9% Trivial Pursuits/Pub Quizzes; though only 2.8% non-cryptic crosswords and 2.3% Scrabble); 9.2% to reading/writing, and learning foreign languages (predominantly reading 6.8%); and 5.8% to academic or niche pursuits (e.g., astronomy, mycology, philately, philology/semiotics and transport enthusiasms). Responses also showed engagement with a wide range of sporting and outdoor activities, whether as participants or spectators (16.9%); and an active interest in the creative arts (7.2%) and music (6.7%).

**Table 8:** **Hobby sectors by expertise, showing % of total responses and % of participants with at least one interest in the sector**.

**Hobby Sector**	**O**	**H**	**S**	**Total**
**(A) % OF RESPONSES IN EACH SECTOR**				
Total number of responses	1291	776	620	2687
Mind games (other than cryptics)	41.0%	42.5%	34.5%	39.9%
Sport and outdoor pursuits (e.g., walking)	18.7%	12.6%	18.5%	16.9%
Reading, writing and languages	7.6%	10.8%	10.5%	9.2%
Social, food and relaxation	7.5%	9.4%	9.4%	8.5%
Active engagement with creative arts	8.1%	7.6%	4.8%	7.2%
Active musical engagement	6.2%	6.4%	7.9%	6.7%
Collectors, niche/academic/IT enthusiasms	5.2%	5.4%	7.6%	5.8%
Home maintenance and gardening	2.9%	3.1%	3.7%	3.2%
Travel	1.4%	1.3%	1.5%	1.4%
Public service/leadership	1.4%	0.8%	1.6%	1.3%
**(B) % PARTICIPANTS INDICATING INVOLVEMENT IN SECTOR**				
Total number of participants	353	197	168	718
Mind games	71.7%	77.2%	65.5%	71.7%
Sport and outdoor pursuits (e.g., walking)	43.6%	37.1%	45.8%	42.3%
Reading, writing and languages	25.8%^*^	37.1%^*^	32.7%	30.5%
Social, food and relaxation	20.4%	26.4%	27.4%	23.7%
Active engagement with creative arts	21.5%	24.9%	16.7%	21.3%
Active musical engagement	18.1%	22.8%	22.6%	20.5%
Collectors, niche/academic/IT enthusiasms	15.0%	16.8%	18.5%	16.3%
Home maintenance and gardening	9.9%	11.7%	13.1%	11.1%
Travel	5.1%	5.1%	5.4%	5.2%
Public service/leadership	5.1%	2.5%	4.8%	4.3%

*,**,*** indicates significance at the 0.05, 0.01, 0.001 level.

Data were then examined by expertise group for each hobby sector to identify differences in respondent participation levels (*n* = 718) for each hobby sector (Table 8B). Findings will be reported in greater detail elsewhere. There was a particularly striking tendency for participants to pursue at least one mind game11 in addition to cryptic crosswords (71.7%), with H showing highest levels (O: *n* = 253, 71.7%, *z* = 0.0; H: *n* = 152, 77.2%, *z* = 0.9; S: *n* = 110, 65.5%, *z* = −1.0). Overall, there were modest differences between the groups in a few sectors: e.g., H and S solvers were more likely to have a hobby involving active musical engagement (O: *n* = 64, 18.1%, *z* = −1.0; H: *n* = 45, 22.8%, *z* = 0.7; S: *n* = 38, 22.6%, *z* = 0.6), but S were less likely to engage in creative arts (O: *n* = 76, 21.5%, *z* = 0.1; H: *n* = 49, 24.9%, *z* = 1.1; S: *n* = 28, 16.7%, *z* = −1.3). However, only the comparison of participation in reading/writing/languages by expertise group was statistically significant [$\chi_{\left(2\right)}^{2}$ = 8.103, *p* = 0.017, Cramer's *V* = 0.106]. Scores were higher among E solvers than O, with H showing particularly high levels of involvement (O: *n* = 91, 25.8%, *z* = −1.6; H: *n* = 73, 37.1%, *z* = 1.7; S: *n* = 55, 32.7%, *z* = 0.5).

#### Musical interest/ability

In addition to the hobbies question, participants were asked to indicate their level of ability in music-making. Responses were given on a 6-point Likert scale (1 = “No interest/ability”; 6 = “Total immersion/professional level”). There were 767 responses to this question (O: *n* = 377; H: *n* = 215; S: *n* = 175). Overall, participants indicated that they had a modest competence in music (*M* = 3.02, *Mdn* = 3). Musical ratings were slightly higher for S participants (O: *M* = 3.00; H: *M* = 2.99; S: *M* = 3.13; all groups *Mdn* = 3). However, the overall comparison failed to achieve statistical significance [*H*_(2)_ = 1.864, *p* = 0.394].

Participants were also asked whether they still participated in musical activities, and whether they sang, played or composed. Responses are summarized in Table 9.

**Table 9:** **Active participation in musical activities at time of survey, by expertise group, in comparison with Arts Council figures for England 2013/14**.

	**O**	**H**	**S**	**Total**	**Arts Council**
Number of responses	377	215	175	767	
Sing	14.3%	10.7%	18.3%	14.2%	3.9%
Play Instrument	21.5%	21.4%	20.0%	21.1%	10.6%
Compose	5.6%	4.7%	4.6%	5.1%	2.6%

Responses were evenly matched across all groups, with the exception of singing, which was particularly high for S (18.3%, *z* = 1.4) and low for H (10.7%, *z* = −1.4). Even so, differences in the distribution of singing participation between groups failed to reach statistical significance [$\chi_{\left(2\right)}^{2}$ = 4.564, *p* = 0.102, Cramer's *V* = 0.077]. Comparison with levels of singing, playing and composing in the general adult English population (DCMS, 2015) suggests that in all three areas the level of active music participation amongst cryptic crossword solvers is markedly above population norms.

#### Hobbies analyzed by RIASEC coding

Finally, hobbies (*n* = 2687) were analyzed by RIASEC coding using the 2-letter RIASEC code (“IR,” “AS” etc.) as supplied by the “Leisure Activities Finder” (Holmberg et al., 1997). Twenty-six of the 30 available codes were used. Over 70% of the hobbies fell into one of 7 categories: as Table 10 indicates, there were no marked differences in the distribution of responses between expertise groups; although H participants engaged least with both personal and team sport, and most with the arts; and S participants engaged least with word-based games and activities.

**Table 10:** **Distribution of hobby responses among RIASEC codes, by expertise**.

**RIASEC code**	**Typical hobbies assigned to code**	**O (%)**	**H (%)**	**S (%)**	**Total (%)**	**Cumulative (%)**
AS	Musical listening and performance; engagement with cultural events, including art galleries, theaters and concerts	10.7	13.0	12.7	11.8	11.8
IE	Board games, strategy computer games, chess	11.7	12.5	10.0	11.5	23.4
SR	Personal fitness, walking, non-team sports	12.3	8.1	13.1	11.3	35.6
IC	Sudoku	10.4	11.7	11.0	10.9	46.9
AI	Word games, code-cracker word puzzles, learning languages, writing, non-cryptics	11.3	9.5	7.4	9.9	55.7
SE	Team sports, sports spectating	9.1	7.3	9.4	8.6	63.1
AC	Reading (fiction; newspapers)	6.1	7.6	7.9	7.0	70.2
RC	Needlework, knitting, baking, woodwork, flower gardening	6.5	5.9	5.0	6.0	76.4
CR	Jigsaws, solitaire, transport enthusiasms	5.0	5.4	4.2	4.9	81.5
IR	Academic (scientific) research and computer programming	3.7	4.5	3.4	3.9	85.5
Other	All other codes (*n* = 16, all < 3% of responses)	13.2	14.3	16.0	14.1	100.0

On a person-specific basis, primary RIASEC hobby codes were then aggregated and averaged to produce an individual primary RIASEC code profile. Results are shown in Table 11.

**Table 11:** **RIASEC analysis of hobby primary codes by expertise group**.

	**R (%)**	**I (%)**	**A (%)**	**S (%)**	**E (%)**	**C (%)**	
O	11.0	26.0	30.2	24.1	0.9	7.7	100
H	10.7	29.6	32.8	17.3^**^	1.0	8.6	100
S	7.7	26.8	29.3	26.3^**^	1.6	8.4	100
Total	10.1	27.2	30.7	22.8	1.1	8.1	100

** indicates significant (p ≤ 0.01) pair-wise comparison between at least two groups: see text.

Once again, the aggregation revealed a very strong tendency across all groups toward “I” activities (27.2%; O: 26.0%; H: 29.6%; S: 26.8%). “A” activities were even more important, and were higher among H solvers than O and S (30.7%; O: 30.2%; H: 32.8%, S: 29.3%): these involved musical, cultural, literary and word-based activities. “S” activities featured prominently for O and S participants, but not for H (22.8%; O: 24.1%; H: 17.3%; S: 26.3%): these tended to be socializing activities involving clubs, sports and family/friends.

Analysis of the distribution of RIASEC hobby scores by expertise was significant only for “S” activities [*H*_(2)_ = 9.221, *p* = 0.010]. Pairwise comparisons were performed as above; adjusted *p-*values are presented. There were only statistically significant differences in scores between H-S, with S showing highest and H lowest scores (*p* = 0.009, *r* = 0.15).

### Motivation in everyday life and crossword solving

#### The “work preference inventory”

Participants were asked to complete the “Work Preference Inventory” (WPI; Amabile et al., 1994), which was “designed as a direct, explicit assessment of individual differences in the degree to which adults perceive themselves to be intrinsically and extrinsically motivated toward what they do” (p. 952). Typical questions include “*To me, success means doing better than other people*” and “*I have to feel that I'm earning something for what I do.”* There were 766 responses (O: *n* = 377; H: *n* = 215; S: *n* = 174) rating each of 29 statements on a 5-point Likert scale (1 = “Completely Disagree”; 5 = “Completely Agree”). Full details of the results obtained will be discussed elsewhere; the current paper presents summarized data only.

Data were analyzed using Amabile et al.'s (1994) 2-factor breakdown, into Intrinsically (IM: *n* = 14) and Extrinsically (EM: *n* = 15) motivated statements, and averaged for each participant within these categories. Overall, respondents showed higher scores on IM items (*M* = 3.63, *Mdn* = 3.64) than EM items (*M* = 2.62, *Mdn* = 2.60), and mean WPI levels were significantly different from the mid-point (3) for both categories [IM: *t*_(765)_ = 32.73, *p* < 0.001, *d* = 1.18; EM: *t*_(765)_ = −18.97, *p* < 0.001, *d* = −0.69]. A Wilcoxon signed-rank test determined that there was a highly statistically significant difference (*Mdn Diff* = 1.04) between responses on IM and EM (*z* = 22.74, *p* < 0.001, *r* = 0.58).

IM increased with expertise (O: *M* = 3.58; H: *M* = 3.67; S: *M* = 3.68) and differences were statistically significant [*H*_(2)_ = 6.42, *p* = 0.040]. Pairwise comparisons were performed as above. These revealed differences in IM between O-S which approached statistical significance (*p* = 0.096, *r* = 0.09). The differences between H-O and H-S were not statistically significant. EM statements were rated most highly by H solvers and least highly by S (O: *M* = 2.60; H: *M* = 2.69; S: *M* = 2.57) but differences were not statistically significant [*H*_(2)_ = 3.12, *p* = 0.21].

#### Motivation for solving crosswords

Participants were also asked to rate 26 statements relating to their motivation for solving cryptic crosswords on a 5-point Likert scale (1 = “Completely Disagree”; 5 = “Completely Agree”). The 26 statements were independently assigned to Intrinsic (IM: *n* = 19) and Extrinsic (EM: *n* = 7) motivational categories by the authors, following the methodology used in the WPI (Amabile et al., 1994). There were 786 responses (O: *n* = 388; H: *n* = 221; S: *n* = 177). Full details of the results obtained will be discussed elsewhere; the current paper presents summarized data only.

Data were analyzed into IM and EM statements (as above), and averaged for each participant within these categories. Overall, respondents again showed higher scores on IM items (*M* = 2.87, *Mdn* = 2.84) than on EM items (*M* = 1.53, *Mdn* = 1.43): see Table 12A. A Wilcoxon signed-rank test determined that there was a highly statistically significant difference (*Mdn Diff* = 1.41) between responses on IM and EM (*z* = 24.23, *p* < 0.001, *r* = 0.61).

**Table 12:** **Intrinsic/Extrinsic crossword solving motivators**.

	**O**	**H**	**S**	**All Groups**
**(A) Overall Means/Mdns**				
Number of respondents	388	221	177	786
Extrinsic Motivation – Mean/(Mdn)	1.32 (1.14)	1.52 (1.43)	2.00 (2.00)	1.53 (1.43)
Intrinsic Motivation – Mean/(Mdn)	2.73 (2.74)	2.96 (3.00)	3.04 (3.05)	2.87 (2.84)
**(B) Highest/Lowest ranked responses**				
**Highest scoring responses: all IM (Mean)**				
1. Enjoy “Penny-Drop Moment”	3.92	3.92	4.07	3.96
2. Cryptics are uniquely satisfying	3.89	4.05	3.91	3.94
3. Mental exercise to keep brain sharp	3.88	3.83	3.85	3.86
4. Makes me smile or laugh	3.79	3.80	3.64	3.76
5. Satisfaction of filled grid	3.46	3.61	3.36	3.48
**Lowest scoring responses: all EM (Mean)**				
22. Social teamwork	1.70	1.51	1.37	1.57
23. Compete against others	1.29	1.63	1.83	1.51
24. Impress bystanders	1.38	1.39	1.38	1.39
25. Do well in tournament	1.11	1.35	2.04	1.38
26. It's my job	1.02	1.06	1.73	1.19
**Other items of interest**				
10. Brain demands cognitive engagement	2.72	3.20	3.36	3.00
14. Enjoy learning new words	2.43	2.56	2.49	2.48

IM increased with expertise (O: *M* = 2.73; H: *M* = 2.96; S: *M* = 3.04) and differences were statistically significant [*H*_(2)_ = 39.28, *p* < 0.001]. Pairwise comparisons were performed as above. These revealed significant differences in IM between O-S (*p* < 0.001, *r* = 0.23) and O-H (*p* < 0.001, *r* = 0.19). The difference between H-S was not statistically significant.

EM increased with expertise (O: *M* = 1.32; H: *M* = 1.52; S: *M* = 2.00) and differences were statistically significant [*H*_(2)_ = 166.85, *p* < 0.001]. Pairwise comparisons were performed as above. These revealed significant differences in EM between O-S, O-H and H-S (all *p* < 0.001; effect sizes O-H *r* = 0.20; O-S *r* = 0.54; H-S *r* = 0.38).

Table 12B shows the 5 highest and 5 lowest ranked responses to the 26 statements (with abbreviated descriptions). All groups rated the “Aha!” or “Penny-Drop Moment” (PDM) as a key motivational factor for solving cryptics; closely allied with this was the statement “*Solving well-written clues gives me a buzz—it makes me smile or laugh out loud*” which was ranked 4th in importance. In a separate question (“*Is your enjoyment of the “penny-drop” moment enhanced if you have had to struggle with the clue?”*) only 11 of the 797 respondents (1.4%) claimed not to have had a PDM when solving cryptics, whereas 634 (79.5%) agreed that it had been strengthened by a stiff challenge. All 5 top-rated statements related to intrinsically motivated reasons, primarily concerned with intellectual challenge and the joyful and satisfying feeling of cracking a clue. There was no statistically significant difference between the groups for any of these statements. The lowest rated statements were all concerned with extrinsically driven reasons such as competition, prestige or collaboration: the median score for all of these questions was 1 across all groups, indicating a rejection of these suggestions.

Respondents were not generally drawn to cryptic crossword solving in order to learn new words (see Table 12B, other items): this suggestion came 14th, and the average response score was significantly lower than the mid-point (3): *t*_(785)_ = −12.02, *p* < 0.001, *d* = −0.43. H solvers were most likely to agree more strongly with this statement (O: *M* = 2.43; H: *M* = 2.56; S: *M* = 2.49), but comparison between expertise groups was not statistically significant [*H*_(2)_ = 1.641, *p* = 0.440].

## Discussion

So far as we are aware, this study is the first to employ a detailed and wide-ranging survey in a performance domain to characterize the nature of expert and non-expert participants across a large number of dimensions. This novel approach proved highly effective, with our results directly influencing subsequent research and providing empirical support for the direction and design of key elements in our studies.

Our findings fully supported the first hypothesis—that cryptic crossword solvers would generally be academically able adults, given the cognitive complexity of the puzzle demands. Over 80% of participants had achieved a university qualification of at least BA/BSc (with nearly 12% of these at doctorate level). This is approximately 8–10 times higher than UK university participation for the most relevant time-period. Furthermore, participants generally pursued cognitively complex post-university careers, with average Holland complexity scores for all groups being indicative of highly-trained, graduate or post-graduate professions. In all group comparisons, the (non-significant) trend was for complexity and academic achievement to be higher for E than for O solvers.

Our second hypothesis—that solvers' education and occupation would predominantly be in scientific or IT-related fields, rather than in language-related fields—was also supported. STEM subjects accounted for over half of university courses, compared to just over a quarter for Wordsmith specialisms. Post-university careers in the STEM and Finance areas continued this trend. Overall, nearly a quarter of all participants worked in IT, rising to almost a third among the S group. Indeed, S participants were significantly more likely to have studied Math and to have worked in the areas of IT or Banking/Accountancy than the other groups. Furthermore, when data were viewed through the prism of RIASEC coding, the code “IRE” (e.g., computing, math, engineering, chemistry) was noticeably prominent and increased with expertise: significantly so, in the case of education. Both education and occupation codings as a whole showed a very strong bias toward the RIASEC code “I,” generally thought to denote analytical, scholarly, scientific and research-oriented individuals; again this was particularly prominent for S solvers. Comparison with US workforce norms indicated that the level of employment in “I” occupations among our survey population was four times greater than might have been expected. Finally, even in their spare time, solvers opted for hobbies which were weighted toward intellectually stimulating activities (“I”); and the logical challenge of Sudoku (RIASEC code “IC”, 9.8% hobby responses) was more popular than word puzzles, languages and writing (RIASEC code “AI”, 7.3% hobby responses). Interestingly, playing Scrabble and solving non-cryptic crosswords were comparatively unpopular hobbies.

Wordsmith skills were thus dwarfed by STEM involvement, but were not entirely irrelevant to our participants. Reading for pleasure was a common hobby, although wherever Wordsmith activities occurred, trends (though non-significant) indicated slightly higher levels for H solvers (e.g., pursuing “A” RIASEC-coded hobbies, following “A” RIASEC-coded careers, studying English at university, aiming to increase vocabulary through crosswords, and reading as a hobby). Conversely, S solvers were less likely than H or O to study English/languages at university and to pursue a Wordsmith career. It is possible, therefore, that word skills are a particular feature of H participation in cryptic solving, but that they do not translate into Super-Expert performance, where coding/analytical aspects of cryptic solving may be more relevant.

The findings relating to the first two hypotheses influenced our research in a number of ways. Firstly, they provided strong corroborative endorsement of the cognitive drivers we hypothesized would be key to solving cryptics. Our data thus appear to confirm that cryptics particularly appeal to academically able, logical and analytical thinkers with strong mathematical/computing aptitude. As Underwood originally suggested, cryptic crossword skill therefore appears to be bound up with code-cracking and problem-solving skills of a quasi-algebraic nature (Underwood et al., 1988). Conversely, lexical ability, although no doubt valuable, does not appear to be a critical discriminator of high expertise among elite solvers. Although tests of lexical breadth and word retrieval did form part of our subsequent research programs, they were therefore not our primary focus: in this we deviated from the Nottingham studies (Underwood et al., 1994; Deihim-Aazami, 1999).

Given the high academic achievement across the entire sample, we hypothesized for our later trials that cognitively straightforward tests of WM load (e.g., simple and complex digit span tasks, or tests of visual short-term memory) would be unlikely to discriminate among groups as effectively as challenging fluid intelligence tasks, which (like cryptics) require the segregation and assembly of multiple task parts and the understanding of their controlling rules (Duncan et al., 2012). Fluid intelligence testing had already been shown not to distinguish between expertise levels in the Nottingham trials (Underwood et al., 1994; Deihim-Aazami, 1999); however, our survey results indicated that the test originally selected in these studies—the AH4 (Heim, 1970), designed for those who ceased education at 18—would have been wholly underpowered for the assessment of such a highly academically qualified population, leading to ceiling effects. A rerun of this comparison using the more appropriate AH5 test (Heim, 1968) was therefore a key priority for our research.

Our third hypothesis, that cryptic crosswords regularly trigger “Aha!” moments and function as a classic type of insight problem through misdirection, was also confirmed. All groups rated the “Aha!” moment as a key motivational reason for solving cryptics, and strongly agreed that its intensity was enhanced by the need to struggle with the clue. Confirmation that insight moments are indeed a key feature of the cryptic experience provided an important research rationale for subsequent phases of our studies, in which we explored individual differences in the ability to resist or resolve deliberate misdirection within our crossword solving population.

The “Aha!” finding also had profound implications for our selection of a representative task to capture the essence of expertise in cryptic solving during lab-based trials. Whereas other similar trials (Deihim-Aazami, 1999; Tuffiash et al., 2007) had followed de Groot's highly influential paradigms involving isolated, briefly presented challenges (de Groot, 1946/1965; Gobet et al., 2004), we felt that this would elicit only meager and comparatively trivial process-tracing data for our research domain, while missing key areas of interest. Indeed, Tuffiash acknowledged similar concerns in his Scrabble study, reporting that “the verbal reports of our SCRABBLE players mainly consisted of strings of candidate solution words” (Tuffiash et al., 2007, p. 129), in response to a one-shot “best-next-move” challenge, restricting their subsequent analysis to a head-count of bingo words, non-bingo words and illegal words. Similarly, Deihim-Aazami reported a high proportion of lean responses to the talk-aloud task (the solving of 37 independent clues without a grid), commenting that “many experts gave incomplete verbalizations and this was due to the simplicity of the clues.” (Deihim-Aazami, 1999, p. 124).

For this reason, in the GECA, we deliberately chose to set our participants the more representative task of solving an entire professionally-commissioned, high-quality cryptic crossword with grid. Performance was video-recorded and transcribed for both verbal and action-based data. In this way, we hoped to exploit the full potential of the think-aloud protocol, capturing a wide range of strategically important ancillary factors such as: chosen solving order of clues; length of time spent in impasse on each clue before moving onto another; frequency of return to an obstinately resistant item; perseveration with an incorrect solution pathway; the antecedents of “Aha!” solution moments; and the use of cross-checking letters as solution prompts. The approach also permitted data capture on the clarity of understanding of clue architecture, frequency of dictionary use, handwritten jottings (such as candidate anagram letters) and the shifting emotional state of our participants (e.g., frustration, triumph, laughter). In this we deviated from the Nottingham studies which had decided on *a priori* grounds that a grid layout was unnecessary since “true expertise is reflected in the ability to tackle the clue and not in whether there are intersecting letters” (Deihim-Aazami, 1999, p. 83). By contrast, we felt a reluctance to impose any such preconceived ideas upon the current study; rather, we preferred to allow the verbal protocol paradigm itself to elicit rich, unconstrained and ecologically valid process-tracing data, and to allow this to drive the identification of key cognitive and metacognitive drivers of high expertise in this domain.

We anticipated (hypothesis four) that solvers would generally enjoy effortful cognitive activity in all spheres of life including work and hobbies, and that this would be an important driver of cryptic crossword participation. This, too, was supported by our findings. Solvers voluntarily chose to engage with intellectually and culturally stimulating activities (music, theater, the arts) coded to “I” and “A” (RIASEC) in their leisure time, and their Need for Cognition scores (Cacioppo et al., 1984) were significantly higher than the test mid-point. The general trend was for enjoyment of effortful thought to increase with expertise, and a number of analyses comparing O and E solvers either achieved or approached statistical significance.

Allied to this were the findings that crossword solving is indeed an intrinsically motivated activity, undertaken for the love of mental stimulation and for personally gratifying insight rewards (hypothesis five). Extrinsically motivated reasons, such as prizes, competitions or impressing others, were not important to our participants, who also showed a significantly more intrinsically than extrinsically motivated profile in relation to their workplace role. We anticipated that practice/engagement levels for both expert and non-expert solvers would consequently be low and relatively unstructured compared to high profile competitive performance areas such as chess, Scrabble and music, where those who aspire to monetary rewards and worldwide prestige must undertake a heavy and inescapable burden of intense practice and effortful learning. This proved the case: time spent solving crosswords each week amounted to only 6–7 h, with no statistical difference between expertise groups. This equates to only 45–60 min per day, or 1–2 crosswords at typical solving speeds. Excluding those who set crosswords professionally, participants in all groups recorded only minimal time spent on other crossword related activity, amounting to approximately 20 min per day. Yet, at the end of several decades of solving, participants had achieved quite different expertise outcomes. Differences in the levels of day-to-day solving activities were therefore not significant, and seemed unlikely to account for performance differences between the solver groups.

Similarly, hypothesis six—that cryptic crossword solving would not normally begin in childhood, in view of the cognitive complexity of the task, but was more likely to have commenced in late teenage years—was also supported. This has important implications for the exploration of practice effects in the domain: unlike many other expertise domains (famously, chess and music) the solving of cryptic crosswords is a hobby chosen voluntarily at the onset of adulthood and pursued primarily for intrinsic enjoyment. Study of the skill acquisition phase and of the role of practice in this domain is therefore refreshingly free from potential confounds such as early childhood practice routines, intense parental pressure or extrinsically rewarded competition circuits. Our subsequent research pursued this line, looking at various aspects of cryptic crossword experience, including the degree of difficulty and the range of crosswords tackled, and the definition and extent—if any—of deliberate practice (Ericsson et al., 1993; Hambrick et al., 2014b; Moxley et al., 2015) in this domain.

## Conclusion

Research into expert performance has traditionally centered upon a limited number of domains and has explored only a small number of aspects such as practice, starting age and WM capacity, based on *a priori* assumptions about the skill-sets required for excellence in the field. Cryptic crosswords have the potential to bring fresh perspectives to the debate: at the highest level of performance, the domain is represented by an elite body of demonstrably top-class practitioners; yet it is atypically unburdened by extreme practice routines, and is motivated by intrinsic rather than highly competitive extrinsic rewards. We have chosen to investigate this promising domain using a new, broad-based paradigm: the *Grounded Expertise Components Approach.*

Our paradigm makes two important modifications to the traditional *Expert-Performance Approach.* First, we argue that although it may be useful to conduct a paper review of hypothesized key cognitive skills in the domain, it is misleading to do this in isolation, without exploring the nature of those who undertake this activity, across a wide number of dimensions. A detailed and open-minded characterization of the target population has proved invaluable in exploring the cryptic crossword domain, and has provided a secure empirical research rationale on which to base subsequent studies. We believe that it is a valid and valuable approach for other performance domains, and that this should be the first task in any such research program. By contrast, under the traditional paradigm, a questionnaire is more usually conducted at the end of the research, and used as a vehicle to capture experience and practice details alone.

One of the strengths of the GECA is thus its ability to suggest productive avenues for future research based on a secure rationale, and this has been the case for UK-style cryptic crosswords. An additional bonus is its ability to throw up interesting and relatively unexpected findings—such as the significant association between left-handedness and professional cryptic crossword setting, the comparatively high level of active musical participation amongst solvers, and the greater proportion of males apparently engaged in the domain, all of which we intend to investigate further. We suggest that—by framing the initial investigation widely—researchers can avoid that circularity of research which captures data only for theoretically predicted elements, and then attempts to restrict the characterization of the activity as a whole to these highly circumscribed results.

Secondly, we argue that care should be taken in choosing a representative lab-based task which enables a full exploration of all facets of performance in the field, exploiting the talk-aloud protocol to its full potential. One-shot, isolated tasks can never reflect the complete range of skills and strategies involved in carrying out the actual task itself, and the aim should be to replicate the demands of the full challenge wherever possible in order to identify key drivers of expertise. Where isolated tasks are chosen as the basis of the EPA procedure there is a risk that investigator preconceptions may lead to unintentional research biases, resulting in the overestimation of the importance of particular niche skills of interest, and the failure to seek or observe potentially key data on other relevant cognitive factors and broader strategic elements.

In conclusion, we agree wholeheartedly with the recent verdict of Hambrick and colleagues: “For scientists, the task now is to develop and test falsifiable theories of expertise that include as many relevant constructs as possible” (Hambrick et al., 2014a, p. 11). The time is therefore right for a more broad-based approach; and we believe that the *Grounded Expertise Components Approach* should play a role in this process.

## Author contributions

KF designed the survey and analyzed data via an Access database. PF and KF reviewed data and agreed coding treatments. KF drafted the article and KF and PF reviewed and finalized it.

### Conflict of Interest Statement

The authors declare that the research was conducted in the absence of any commercial or financial relationships that could be construed as a potential conflict of interest.

## Footnotes

^1^Total number of individuals submitting entries in each year ranges from 1500 to 3300. See further the official *Listener* site www.listenercrossword.com.

^2^http://www.piemag.com/about/.

^3^The *Times* National Crossword Championship (TNCC) is the only UK competitive forum for speed-solving, and the prize is currently £1000.

^4^As confirmed by Derek Harrison, website owner, e-mail dated 18/9/09.

^5^Data supplied by Chris Lear, Magpie editorial team member, e-mail dated 25/9/09.

## References

[B1] Aarons D. (2012). Jokes and the Linguistic Mind. London: Routledge.

[B2] Almond N. M. (2010). Use-it-or-Lose-it: Investigating the Cognitive Reserve Hypothesis and Use-Dependency Theory. Ph.D. Leeds.

[B3] Amabile T. M. Hill K. G. Hennessey B. A. Tighe E. M. (1994). The Work Preference Inventory: assessing intrinsic and extrinsic motivational orientations. J. Pers. Soc. Psychol. 66, 950–967. 10.1037/0022-3514.66.5.950 8014837

[B4] Balfour S. (2004, December 6). The Last Word. The Guardian.

[B5] Biddlecombe P. (2011, December 6). Yet Another Guide to Cryptic Crosswords (YAGCC) - Puzzle Sources (Newspapers Magazines). Available online at: http://www.biddlecombe.demon.co.uk/yagcc/YAGCC5.html.

[B6] Biddlecombe P. (2012, October 27). The Times Crossword Championship - Unofficial Information. Available online at: http://www.biddlecombe.demon.co.uk/timescmp.html.

[B7] Bilalić M. McLeod P. Gobet F. (2007). Does chess need intelligence? - A study with young chess players. Intelligence 35, 457–470. 10.1016/j.intell.2006.09.005

[B8] Bolton P. (2012). Education: Historical Statistics. House of Commons Library. London. Available online at: http://researchbriefings.parliament.uk/ResearchBriefing/Summary/SN04252

[B9] Brandreth G. (2013, November 23). Why We all Love a Crossword. The Telegraph. Available online at: http://www.telegraph.co.uk/lifestyle/10469311/Why-we-all-love-a-crossword.html.

[B10] Cacioppo J. T. Petty R. E. Kao C. E. (1984). The efficient assessment of need for cognition. J. Pers. Assess. 48, 306–307. 10.1207/s15327752jpa4803_13 16367530

[B11] Campitelli G. (2015). Answering research questions without calculating the mean. Front. Psychol. 6:1379. 10.3389/fpsyg.2015.01379 26441754 PMC4562244

[B12] Campitelli G. Gobet F. (2010). Herbert Simon's decision-making approach: investigation of cognitive processes in experts. Rev. Gen. Psychol. 14, 354–364. 10.1037/a0021256

[B13] Carroll J. B. (1982). The measurement of intelligence, in Handbook of Human Intelligence, ed Sternberg R. J. (Cambridge, MA: Cambridge University Press), 29–120.

[B14] Cattell R. B. (1963). Theory of fluid and crystallized intelligence: a critical experiment. J. Educ. Psychol. 54, 1–21. 10.1037/h0046743 6043849

[B15] Chase W. Simon H. A. (1973). Perception in chess. Cogn. Psychol. 4, 55–81. 10.1016/0010-0285(73)90004-2

[B16] Chi M. T. H. (2006). Two approaches to the study of experts' characteristics, Ch. 2 in The Cambridge Handbook of Expertise and Expert Performance, eds Ericsson K. A. Charness N. Hoffman R. R. Feltovich P. (Cambridge: Cambridge University Press), 21–30. 10.1017/CBO9780511816796.002

[B17] Chi M. T. H. Feltovich P. J. Glaser R. (1981). Categorization and representation of physics problems by experts and novices. Cogn. Sci. 5, 121–152. 10.1207/s15516709cog0502_2

[B18] Coffey S. (1998). Linguistic Aspects of the Cryptic Crossword. English Today, 53, 14–18. 10.1017/S0266078400000663

[B19] Connor A. (2012, May 31). Crossword blog: Crosswords for beginners: which papers are easiest?, The Guardian. Available online at: http://www.theguardian.com/crosswords/crossword-blog/2012/may/31/crosswords-for-beginners-which-papers-are-easiest.

[B20] Connor A. (2013, January 31). Crossword blog: Meet the Setter – Philistine. *The Guardian* Available online at: http://www.theguardian.com/crosswords/crossword-blog/2013/jan/31/crossword-blog-meet-the-setter-philistine.

[B21] Connor A. (2014). Two Girls, One on Each Knee (7). London: Penguin Books.

[B22] Davidson J. E. (2003). Insights about insightful problem solving, in The Psychology of Problem Solving, eds Davidson J. E. Sternberg R. J. (New York, NY: Cambridge University Press), 149–175. 10.1007/s11427-011-4233-3

[B23] DCMS (2015). Taking Part 2013/14, Focus on: Art forms. Department for Culture, Media and Sport, UK. Available online at: https://www.gov.uk/government/uploads/system/uploads/attachment_data/file/413046/Y9_Arts_Short_Story.pdf

[B24] Deary I. J. Smith P. (2004). Intelligence research and assessment in the United Kingdom, in International Handbook of Intelligence, ed Sternberg R. J. (Cambridge: Cambridge University Press), 1–48. 10.1210/jc.2015-2318

[B25] de Cuevas J. (2004). Survey of PuzzleCrypt.com Members. Available online at: http://www.puzzlecrypt.com/public/z-report.pdf

[B26] de Groot A. (1946/1965). Thought Choice in Chess. The Hague: Mouton Publishers.

[B27] Deihim-Aazami C. (1999). Cognitive Expertise in Solving Crossword Puzzles. Ph.D. University of Nottingham.

[B28] Detterman D. K. (2014). Introduction to the intelligence special issue on the development of expertise: is ability necessary? Intelligence 45, 1–5. 10.1016/j.intell.2014.02.004

[B29] DeYoung C. G. Peterson J. B. Flanders J. L. (2008). Cognitive abilities involved in insight problem solving: an individual differences model. Creat. Res. J. 20, 278–290. 10.1080/10400410802278719

[B30] Duncan J. Schramm M. Thompson R. Dumontheil I. (2012). Task rules, working memory, and fluid intelligence. Psychon. Bull. Rev. 19, 864–870. 10.3758/s13423-012-0225-y 22806448 PMC3456922

[B31] Dunn O. J. (1964). Multiple comparisons using rank sums. Technometrics 6, 241–252. 10.1080/00401706.1964.10490181

[B32] Ericsson K. A. (2000). Expertise in interpreting: an expert-performance perspective. Interpreting 5, 187–220. 10.1075/intp.5.2.08eri

[B33] Ericsson K. A. (2006). Protocol analysis and expert thought: concurrent verbalizations of thinking during experts' performance on representative tasks, in Cambridge Handbook of Expertise and Expert Performance, eds Ericsson K. A. Charness N. Feltovich P. Hoffman R. R. (Cambridge: Cambridge University Press), 223–242.

[B34] Ericsson K. A. Krampe R. T. Tesch-Römer C. (1993). The role of deliberate practice in the acquisition of expert performance. Psychol. Rev. 100, 363–406. 10.1037/0033-295X.100.3.363

[B35] Ericsson K. A. Smith J. (1991). Toward a General Theory of Expertise: Prospects and Limits. Cambridge, UK: Cambridge University Press.

[B36] Ericsson K. A. Towne T. J. (2010). Expertise. Wiley Interdisc. Rev. Cogn. Sci. 1, 404–416. 10.1002/wcs.47 26271380

[B37] Ericsson K. A. Ward P. (2007). Capturing the naturally occurring superior performance of experts in the laboratory: toward a science of expert and exceptional performance. Curr. Dir. Psychol. Sci. 16, 346–350. 10.1111/j.1467-8721.2007.00533.x

[B38] Ericsson K. A. Williams A. M. (2007). Capturing naturally occurring superior performance in the laboratory: translational research on expert performance. J. Exp. Psychol. Appl. 13, 115–123. 10.1037/1076-898X.13.3.115 17924797

[B39] Fine P. A. Friedlander K. J. (2010). A study of expert and non-expert cryptic crossword solvers, Paper presented at the 27th BPS Cognitive Psychology Conference (Cardiff).

[B40] Forshaw M. (1994). Expertise and Ageing: The Crossword Puzzle Paradigm. Ph.D. University of Manchester.

[B41] Friedlander K. J. Fine P. A. (2009). Expertise in cryptic crossword performance: an exploratory survey, in *Proceedings of the International Symposium on Performance Science, Auckland,* eds Williamon A. Pretty S. Buck R. (Utrecht: European Association of Conservatoires (AEC)), 279–284.

[B42] Gilbert V. (2001). The ‘Daily Telegraph’: How to Crack a Cryptic Crossword, 2nd Edn. London, Basingstoke, Oxford: Pan.

[B43] Gilhooly K. J. Green C. (1996). Protocol analysis: theoretical background, in Handbook of Qualitative Research Methods for Psychology and the Social Sciences, ed Richardson J. T. E. (Leicester: BPS Books), 43–54.

[B44] Gobet F. Campitelli G. (2007). The role of domain-specific practice, handedness, and starting age in chess. Dev. Psychol. 43, 159–172. 10.1037/0012-1649.43.1.159 17201516

[B45] Gobet F. Charness N. (2006). Expertise in chess, in Cambridge Handbook on Expertise and Expert Performance, eds Ericsson K. A. Charness N. Feltovich P. Hoffman R. R. (Cambridge; New York: Cambridge University Press), 523–538. 10.3389/fnhum.2013.00825

[B46] Gobet F. Ereku M. H. (2014). Checkmate to deliberate practice: the case of Magnus Carlsen. Front. Psychol. 5:878. 10.3389/fpsyg.2014.00878 25177304 PMC4132259

[B47] Gobet F. Retschitzki J. De Voogt A. (2004). Moves in Mind: The Psychology of Board Games: Hove: Psychology Press.

[B48] Gottfredson G. D. Holland J. L. (1996). Dictionary of Holland Occupational Codes, 3rd Edn. Odessa, FL: Psychological Assessment Research Inc.

[B49] Gov.UK (2015). Apprenticeships, 14 to 19 education and training for work: Compare Different Qualifications. Available online at: https://www.gov.uk/what-different-qualification-levels-mean/compare-different-qualification-levels

[B50] Grabner R. H. (2014). The role of intelligence for performance in the prototypical expertise domain of chess. Intelligence 45, 26–33. 10.1016/j.intell.2013.07.023

[B51] Grabner R. H. Stern E. Neubauer A. C. (2007). Individual differences in chess expertise: a psychometric investigation. Acta Psychol. 124, 398–420. 10.1016/j.actpsy.2006.07.008 16942740

[B52] Green C. Gilhooly K. J. (1996). The practical use of protocol analysis: promises and pitfalls, in Handbook of Qualitative Research Methods for Psychology and the Social Sciences, ed J. T. E. Richardson (Leicester: BPS Books).

[B53] Greer B. (2001). How to Do ‘The Times' Crossword. London: HarperCollins.

[B54] Hambrick D. Z. Altmann E. M. Oswald F. L. Meinz E. J. Gobet F. (2014a). Facing facts about deliberate practice. Front. Psychol. 5:751. 10.3389/fpsyg.2014.00751 25101023 PMC4101876

[B55] Hambrick D. Z. Macnamara B. N. Campitelli G. Ullén F. Mosing M. A. (2016). Beyond born versus made: a new look at expertise. Psychol. Learn. Motiv. 64, 1–55. 10.1016/bs.plm.2015.09.001

[B56] Hambrick D. Z. Oswald F. L. Altmann E. M. Meinz E. J. Gobet F. Campitelli G. (2014b). Deliberate practice: is that all it takes to become an expert? Intelligence 45, 34–45. 10.1016/j.intell.2013.04.001

[B57] Hambrick D. Z. Salthouse T. A. Meinz E. J. (1999). Predictors of crossword puzzle proficiency and moderators of age–cognition relations. J. Exp. Psychol. 128, 131–164. 10.1037/0096-3445.128.2.131 10406103

[B58] Harrison D. (n.d.). The Crossword Centre. Available online at: http://www.crossword.org.uk/.

[B59] Heim A. W. (1968). AH5 Group Test of High-grade Intelligence: Manual. Revised Edition. Slough: National Foundation for Educational Research.

[B60] Heim A. W. (1970). AH4 Group Test of General Intelligence: Manual. Slough: National Foundation for Educational Research.

[B61] Hicks K. L. Harrison T. L. Engle R. W. (2015). Wonderlic, working memory capacity, and fluid intelligence. Intelligence 50, 186–195. 10.1016/j.intell.2015.03.005

[B62] Holmberg K. Rosen D. Holland J. L. (1997). The Leisure Activities Finder, SDS Form R, 2nd Edn. Odessa, FL: Psychological Assessment Resources, Inc.

[B63] Horn J. L. Cattell R. B. (1967). Age differences in fluid and crystallized intelligence. Acta Psychol. 26, 107–129. 10.1016/0001-6918(67)90011-X 6037305

[B64] Kane M. J. Hambrick D. Z. Conway A. R. A. (2005). Working memory capacity and fluid intelligence are strongly related constructs: comment on Ackerman, Beier, and Boyle (2005). Psychol. Bull. 131, 66–71. 10.1037/0033-2909.131.1.66 15631552

[B65] Lancaster C. (2005). The Survey of Crossword Centre Subscribers. Available online at: http://www.crossword.org.uk/Survey.pdf

[B66] Lewis M. B. (2006). Eye-witnesses should not do cryptic crosswords prior to identity parades. Perception 35, 1433–1436. 10.1068/p5666 17214386

[B67] Manley D. (2014). Chambers Crossword Manual, 5th Revised Edn. London: Chambers.

[B68] McClain M.-C. Reardon R. C. (2015). The US Workforce from 1960 to 2010: A RIASEC View. The Professional Counselor 5, 1–14. 10.15241/rcr.5.1.1

[B69] Minati L. Sigala N. (2013). Effective connectivity reveals strategy differences in an expert calculator. PLoS ONE 8:e73746. 10.1371/journal.pone.0073746 24086291 PMC3781167

[B70] Moorey T. (2009). Cryptic Crosswords: Clue Types and Indicators. Available online at: http://www.timmoorey.info/pdfs/clue_types.pdf

[B71] Moxley J. H. Ericsson K. A. Scheiner A. Tuffiash M. (2015). The effects of experience and disuse on crossword solving. Appl. Cogn. Psychol. 29, 73–80. 10.1002/acp.3075

[B72] Nauta M. M. (2010). The development, evolution, and status of Holland's theory of vocational personalities: reflections and future directions for counseling psychology. J. Couns. Psychol. 57, 11–22. 10.1037/a0018213 21133557

[B73] Nickerson R. S. (1977). “Crossword puzzles and lexical memory, in Attention and Performance VI: Proceedings of the Sixth International Symposium on Attention and Performance, Stockholm, July 28-August 1, 1975, ed S. Dornič (Hillsdale, NJ: Lawrence Erlbaum), 699–718.

[B74] Nickerson R. S. (2011). Five down, Absquatulated: crossword puzzle clues to how the mind works. Psychon. Bull. Rev. 18, 217–241. 10.3758/s13423-011-0069-x 21347878

[B75] Pretz J. E. Naples A. J. Sternberg R. J. (2003). Recognizing, defining and representing problems, in The Psychology of Problem Solving, eds Davidson J. E. Sternberg R. J. (New York, NY: Cambridge University Press), 3–30. 10.1071/AH15189

[B76] Reardon R. C. Bullock E. E. Meyer K. E. (2007). A Holland perspective on the US workforce from 1960 to 2000. Career Dev. Q. 55, 262–274. 10.1002/j.2161-0045.2007.tb00082.x

[B77] Romano M. (2006). Crossworld: One Man's Journey into America's Crossword Obsession. New York, NY: Broadway Books.

[B78] Schulman A. (1996). The art of the puzzler, in Cognitive Ecology: Handbook of Perception and Cognition, Vol. 16, 2nd Edn., eds Friedman M. P. Carterette E. C. (San Diego, CA: Academic Press), 293–321. 10.1016/B978-012161966-4/50009-8

[B79] Shipstead Z. Lindsey D. R. B. Marshall R. L. Engle R. W. (2014). The mechanisms of working memory capacity: primary memory, secondary memory, and attention control. J. Mem. Lang. 72, 116–141. 10.1016/j.jml.2014.01.004

[B80] Shortz W. (2001). How to Solve the New York Times Crossword Puzzle. New York Times Magazine. Available online at: http://www.nytimes.com/2001/04/08/magazine/08PUZZLE.html

[B81] Simon (2004). The cryptic crossword puzzle as a useful analogue teaching programming, Paper presented at the ACM International Conference Series ACE ‘04: The Sixth Australasian Conference on Computing Education (Dunedin).

[B82] Singh S. (1999). The Code Book: The Science of Secrecy from Ancient Egypt to Quantum Cryptography, 2nd Edn. London: Fourth Estate Ltd.

[B83] Stephenson H. (2007). Secrets of the Setters: How to Solve the ‘Guardian’ Crossword. London: Guardian Books.

[B84] Sutherland D. (2012). Solving Cryptic Crosswords for Dummies. Milton, QLD: Wiley Publishing Australia Pty Ltd.

[B85] Toma M. Halpern D. F. Berger D. E. (2014). Cognitive abilities of elite nationally ranked SCRABBLE and crossword experts. Appl. Cogn. Psychol. 28, 727–737. 10.1002/acp.3059

[B86] Tuffiash M. Roring R. W. Ericsson K. A. (2007). Expert performance in SCRABBLE: implications for the study of the structure and acquisition of complex skills. J. Exp. Psychol. Appl. 13, 124–134. 10.1037/1076-898X.13.3.124 17924798

[B87] Underwood G. Deihim C. Batt V. (1994). Expert performance in solving word puzzles: from retrieval cues to crossword clues. Appl. Cogn. Psychol. 8, 531–548. 10.1002/acp.2350080602

[B88] Underwood G. MacKeith J. Everatt J. (1988). Individual differences in reading skill and lexical memory: the case of the crossword puzzle expert, in Practical Aspects of Memory: Current Research and Issues, Vol. 2: Clinical and Educational Implications, eds Gruneberg M. M. Morris P. E. Sykes R. N. (Chichester: Wiley), 301–308.

[B89] von Stumm S. Ackerman P. L. (2013). Investment and intellect: a review and meta-analysis. Psychol. Bull. 139, 841–869. 10.1037/a0030746 23231531

[B90] Winder B. C. (1993). Intelligence and Expertise in the Elderly. M.Sc. University of Manchester.

