# Supplementary material for: The Grounded Expertise Components Approach in the Novel Area of Cryptic Crossword Solving
Source: Front Psychol. 2016 May 3;7:567. doi: 10.3389/fpsyg.2016.00567 (PMC4853387; doi:10.3389/fpsyg.2016.00567)
Supplement: Supplementary file 1 [file Table1.docx]

**The *Grounded* *Expertise Components Approach* in the novel area of cryptic crossword solving: Supplementary Material**

**Kathryn J. Friedlander^1^*, Philip A. Fine^1^**

^1^Department of Psychology, University of Buckingham, Buckingham, Buckinghamshire, England

*** Correspondence:** Dr. Kathryn Friedlander, Department of Psychology, School of Science and Postgraduate Medicine, University of Buckingham, Buckingham, MK18 1EG, England

kathryn.friedlander@buckingham.ac.uk

**APPENDIX A**

Educational subject fields (*n=*17) and their mapping to educational sector (*n=*6):

| **Main subject field** | **Main subject sector** |
| --- | --- |
| Arts | Arts & Design |
| Design (e.g. Architecture) | Arts & Design |
| Business Studies (including Accountancy) | Business |
| Human Science (e.g. Anthropology, Geography, History) | Human Studies |
| Social Science (e.g. Sociology, PPE, Economics, Law) | Human Studies |
| Engineering/Construction | STEM |
| Mathematics | STEM |
| Medical Science | STEM |
| Natural Science (e.g. Biology, Psychology) | STEM |
| Physical Science | STEM |
| Science (general) | STEM |
| Technology | STEM |
| Languages | Wordsmiths |
| Literature | Wordsmiths |
| Media Studies | Wordsmiths |
| Philosophy/Religion | Wordsmiths |
| Other | Other |

**APPENDIX B**

Occupational fields (*n=*23) and their mapping to occupational sectors (*n=*7):

| **Occupation field** | **Occupation sector** |
| --- | --- |
| Actuarial/Economics | Finance |
| Banking/Accountancy | Finance |
| Engineering | STEM |
| Math/Stats | STEM |
| Medical Science | STEM |
| Science | STEM |
| Technology | STEM |
| Admin/Management | Office/Business |
| Business/Consultancy | Office/Business |
| Legal Work | Office/Business |
| Social Work | Office/Business |
| Creative/Media | Wordsmiths |
| Languages | Wordsmiths |
| Spiritual/Philosophy | Wordsmiths |
| Creative/Performance | Performance |
| Building & Construction | Manual |
| Farming/Manual | Manual |
| Armed Forces and Emergency Servs | Other |
| Investigative/Intelligence* | Other |
| Crossword compiler (full time)* | Other |
| Human Studies (academics)* | Other |
| Other Pedagogy (subject not identified)** | Other |
| Other*** | Other |

* All categories have fewer than 10 responses

** Occupation stated as ‘Teacher/Head Teacher’ with no specialism

*** Declined to answer/never employed
